# Covariant Approach to the Geometric Dilution of Precision

**DOI:** 10.3390/s26154746

**Published:** 2026-07-26

**Authors:** Ramón Serrano Montesinos, Joan Josep Ferrando, Juan Antonio Morales-Lladosa

**Affiliations:** 1 Departament d’Astronomia i Astrofísica, Universitat de València, 46100 Burjassot, València, Spain; joan.ferrando@uv.es (J.J.F.); antonio.morales@uv.es (J.A.M.-L.); 2 Observatori Astronòmic, Universitat de València, 46980 Paterna, València, Spain

**Keywords:** relativistic positioning systems, frequency geometric dilution of precision, covariant approach

## Abstract

A covariant formulation of the Geometric Dilution of Precision (GDOP) matrix is presented in the framework of a Relativistic Positioning System (RPS). By including the receiver–emitter frequency ratios, the Frequency Geometric Dilution of Precision (FGDOP) scalar is computed in terms of observable quantities, the received frequencies and the angular separation between pairs of emitters in view. Some required concepts are first introduced: the FGDOP matrix and the Gram matrix associated to *k* light-like vectors. From the tensor form of the FGDOP matrix and its trace, a closed form of the FGDOP scalar is obtained, extending previous matrix calculations. Clarifying computations for symmetric emitter configurations are presented. The geometric interpretation of the GDOP scalar in terms of volumes and areas defined by the relative position of the emitters on the unit celestial sphere of the user is also recovered.

## 1. Introduction

The term Geometric Dilution of Precision (GDOP) is widely used in the context of Global Navigation Satellite Systems (GNSSs). It refers to a matrix constructed from the positions of the satellites on the sky, the GDOP matrix, and to the square root of its trace, the so-called GDOP scalar. The latter is a dimensionless metric indicator of the goodness in the determination of the user location from the geometry of the satellite configuration in view, assuming a simple stochastic model [1,2,3].

More specifically, the classical positioning approach usually applies the least squares method to minimize the differences between modelled emitter–receiver distances and measured pseudoranges (travel times multiplied by the signal velocity). For *k* satellites in view at the user location, the GDOP matrix is the 4 × 4 matrix defined as the inverse of $H^{T}\times H$, where $H^{T}$ denotes the transpose of *H*, the *k* × 4 matrix whose *A*th row is $\left(1,{\vec{n}}_{A}\right)$, with ${\vec{n}}_{A}=\left(n_{A_{x}},n_{A_{y}},n_{A_{z}}\right)$ the unit vector along the line of sight of the *A*-emitter (A=1,2,…,k) expressed in Cartesian coordinates [1,2,3].

Then, under the hypothesis of unbiased and independent measurement errors, the root mean square error of the estimated user location is directly proportional to the GDOP scalar, the proportionality factor being the (square root of the) variance of the measurements. Therefore, the lower the value of the GDOP scalar, the more favourable the satellite configuration will be to attain an accurate user location and dismiss the consequences of measurement errors. High GDOP scalar values are commonly associated to bad or degenerate satellite distributions unfavourable to an effective user localization.

Furthermore, as shown in [4], when the stochastic model for the pseudorange measurements is supposed to be Gaussian, the Cramér–Rao lower bound in estimating the user position and clock offset is reached by the GDOP matrix. In fact, when dealing with Doppler-aided approaches to positioning [5,6], the estimation of errors also involves the transmitted frequencies. Generally, in Doppler-aided positioning, the Cramér–Rao lower bound is not reached by the covariance matrix formed with the receiver’s position, clock bias, velocity, and bias ratio (if it considered) when applying the usual least squares minimization method.

The GDOP terminology also applies in other location situations, for instance, to guide unmanned vehicles with precision. In this case the GDOP matrix involves the unit vectors pointing towards the vehicle from a station network or a set of spatially separated sensors [7,8].

The algebraic approach to GDOP concepts was analysed and discussed by various authors as soon as the pioneering Global Positioning System (GPS) project was announced [9,10,11,12,13,14]. The GDOP scalar has been expressed in terms of tetrahedron volumes and triangle surfaces constructed from the unit vectors along the direction of the received signals from the emitters to the user [15,16,17]. As proposed in [18,19], the (weighted and non-weighted) GDOP scalar can be computed from the traces of powers of the so-called geometric matrix, which is explicitly carried out in the present work.

By selecting favourable sets of satellites in view, collaboration between current GNSSs should ensure positioning accuracy for a passive network of receptors. The starting point for satellite selection criteria is the GDOP computation or their variants (weighted GDOP) with additional information from a network of satellites other than the sky configuration. In [8] the authors combine GDOP and weighted GDOP computations and near-real-time observation accuracy as a satellite selection method improving other methods based on signal-to-noise ratios. In this paper, we propose a new GDOP study, a fully relativistic one which introduces the received–emitted frequency ratio as additional (non-geometric) system information. Formally, this frequency ratio can operate as a traditional weight in extended GDOP computations, informing us about the effect of Doppler shifts on the degree of positioning precision. Nevertheless, the GDOP approach presented here differs from the classical one in the sense that the theory of relativity is fundamental to introduce, justify, and interpret the concepts involved. The frequency-weighted extended GDOP is a useful tool for improving existing satellite selection methods, either by numerically implementing the covariant Equation (1) given in Section 2 or the geometric Equation (84) given in Section 6.

Nevertheless, the primordial objective of this work is to adapt the current classical GDOP formalism to the theory of Relativistic Positioning Systems (RPSs). In fact, the determination of the user location based on pseudorange measurements by common GNSS receivers [1,3] does not take into account frequency measurements, which are otherwise necessary to determine the user velocity with high accuracy or the position in unfavourable situations [6]. In this regard, the deployment of Low Earth Orbit (LEO) satellites has recently motivated the related notion of Doppler GDOP (DGDOP) [20,21,22]. A comparative account of algorithms used to estimate the user localization in Doppler-aided GNSSs can be found in [23].

The essential ingredients of a RPS are four emitter world-lines broadcasting their respective proper times (or any other time scale) by means of electromagnetic signals. The set of four broadcast times are the emission coordinates of the RPS, which are natural coordinates numbering the emitters’ future-oriented null cones [24]. Emission coordinates admit Newtonian analogous constructions broadcasting sound signals [25]. In [26] it was outlined that in the region where a RPS operates, any event knows its own coordinates immediately (that is, without delay); in addition, the physical realization of a RPS does not require prior knowledge of the gravitational field and can therefore operate in any space-time. That is, at any space-time region, the realization of a RPS allows us to construct a primary reference system, opening the possibility of performing gravimetry, as shown in two-dimensional Lorentzian space-times [26,27,28]. The idea to develop gravimetry from the perspective of RPS theory [29] has motivated an extended classification of the properties required for a RPS [30,31]. On the other hand, emission coordinates have also been studied in [32] using the term GPS coordinates previously introduced in [33,34,35].

For an account of the genesis and current status of RPS theory see [29,33,36,37]. The main ambitious objective of RPS theory is to construct a truly relativistic laboratory with full self-capabilities to perform gravimetry in an unknown space-time region [37]. The interest of RPS theory in GNSS was stressed in [24,33]; it pursues the construction of a global reference system, primarily based on the sky sector (satellites), providing accurate positioning and time localization of the actions of control stations. The transformation from emission to inertial coordinates [38] provides the location of the user in flat space-time, once the emission coordinate domains have been analysed in relation to the bifurcation problem [39,40,41,42]. Numerical implementation of this transformation in GNSS systems has been proposed in [43,44,45,46,47]. Numerical positioning simulations in Schwarzschild and perturbed Schwarzschild space-time have been performed in [36,48].

To attain a relativistic formulation of the GDOP, as an alternative to the Newtonian conception on which the current notion is rooted, some aspects have to be investigated further. The search for a fully relativistic treatment of the GDOP must consider at least two main tasks: (i) to establish a covariant GDOP formalism, based on relativistic terminology, and (ii) to develop a 3 + 1 formulation for the GDOP, relative to an arbitrary observer, within the framework of RPS theory [41]. The present work is mainly focused on the first task (i). The results achieved here pave the way to develop the second and complementary task (ii), which is discussed in [49].

This paper is organized in steps to reach formula (1) advanced in Section 2, which allows us to evaluate the so-called Frequency GDOP (FGDOP) scalar from observable quantities (the receiver–emitter frequency ratios and the angular separation between pairs of emitters). The required concepts are introduced in due time: The notion of emission coordinates (of the broadcast proper times) and its gradients are explained in Section 3, showing the relation between the GDOP and the Jacobian determinant of the transformation from inertial to emission coordinates. The Frequency Geometric Matrix *G* associated with *k* emitters is introduced in Section 4 and the Gram matrix G of *k* null vectors in Section 5. From the tensor form of the FGDOP matrix $G^{-1}$ and its trace, the FGDOP covariant Formula (1) is obtained in Section 6 and applied to symmetric emitter configurations in Section 7. Finally, Section 8 summarizes the results and previews more work in progress. Three appendices have been reserved for results whose proof is more involved: Appendix A and Appendix B present the proofs of Propositions 3 and 4, respectively, and Appendix C is devoted to the detailed proof of Proposition 7.

At first glance, the contents and terminology of the manuscript may appear unfamiliar to a reader not specialized in Relativity. Accommodating for this possibility, we decided from the outset to present the results in the form of propositions distributed throughout the manuscript. We believe that this is the best way to facilitate reading: presenting a concise summary of interrelated propositions, while reserving the detailed proofs for the appendices. Although it is not a widespread practice in engineering-oriented journals, it can be found in certain technical publications (cf. [50]).

## 2. Advancing the FGDOP Formula and the Notation Used

In this work we present a covariant formulation of the Frequency Geometric Dilution of Precision (FGDOP) matrix in the framework of a Relativistic Positioning System (RPS). The *FGDOP* scalar is computed in terms of observable quantities, the frequency ratios, $f_{A}$, and the mutual angles, $\theta_{AB}$ between the *k* emitters, from this formula

(1) $$FGDOP=\sqrt{\frac{\frac{\lambda }{3}+2\Sigma -K\sigma }{K\frac{\lambda }{3}+S\frac{\sigma }{2}-\Lambda }}.$$

with *K*, *σ*, *λ*, *S*, Σ, and Λ given by

$$\begin{array}{cccc} K & \equiv \sum_{A=1}^{k}f_{A}^{2}\equiv \left(f,f\right)\equiv f^{2}, & \;S & \equiv 2\sum_{A<B}^{k}f_{A}f_{B}G_{AB}\equiv G\left(f,f\right),\\ \sigma & \equiv 2\sum_{A<B}^{k}{\left(G_{AB}\right)}^{2}=\mathrm{tr}G^{2}, & \;\Sigma & \equiv \sum_{A,B=1}^{k}f_{A}f_{B}{\left(G^{2}\right)}_{AB}\equiv G^{2}\left(f,f\right),\\ \lambda & \equiv \sum_{A,B,C=1}^{k}G_{AB}G_{BC}G_{CA}=\mathrm{tr}G^{3}, & \;\Lambda & \equiv \sum_{A,B=1}^{k}f_{A}f_{B}{\left(G^{3}\right)}_{AB},\equiv G^{3}\left(f,f\right), \end{array}$$

where $G_{AB}$ is the Gram matrix, whose entries are the scalar products of *k* future-oriented null vectors, ${\left\{l_{A}\right\}}_{A=1}^{k}$, that is,

$$G_{AB}\equiv g\left(l_{A},l_{B}\right)\equiv l_{A}\cdot l_{B}=f_{A}f_{B}\left(-1+\cos\theta_{AB}\right)=-2f_{A}f_{B}\sin^{2}\frac{\theta_{AB}}{2},\;A,B=1,\ldots ,k,$$

with $0\leq \theta_{AB}\leq \pi$, and denoting as $f\equiv (f_{1},f_{2},\ldots ,f_{k})$ the ordered *k*-tuple of frequency ratios. If *u* is the user four-velocity ($u^{2}=-1$), then $l_{A}=f_{A}\left(u+n_{A}\right)$, with $n_{A}$ being the unit vector along the propagation of the signal going from the *A* emitter to the user (that is, $-n_{A}$ is the unit vector on the line of sight, from the user to the *A* emitter). The signature of the space-time metric *g* is taken as (−+++) and the speed of light in vacuum *c* is taken as c=1.

Regarding Equation (1), note that the sum of quaternary products of the involved Gram matrix elements is expressed as sums of ternary products at most. The explicit relationship is shown in Equation (69).

In GNSSs, for each satellite, there is a transmission time lag that contributes to the delay between the transmission and reception of the signal. This is the physical motivation for introducing the frequency ratio $f_{A}$ given by Equation (10). The frequency ratio takes into account the Doppler shift between the user and each satellite and is incorporated into the FGDOP concept.

## 3. Emission Coordinates

As already said, emission coordinates are physically realized by four electromagnetic signals broadcasting the proper times of the four clocks of a minimal RPS (a RPS constituted by four emitters).

The locus of space-time events with a constant proper time, say $\tau^{A}=constant$, A=1,…,4, is a null hypersurface in vacuum, where electromagnetic signals propagate at the velocity of light. For this reason, the variations (or covectors) $d\tau^{A}$ are restricted by the null constraint $(d\tau^{A},d\tau^{A})=0$, and consequently, in emission coordinates, the contravariant components $g^{AB}$ of the space-time metric *g* are given by the following:

(2) $$g^{AB}=g\left(d\tau^{A},d\tau^{B}\right)=\left(\begin{array}{cccc} 0 & g^{12} & g^{13} & g^{14}\\ g^{21} & 0 & g^{23} & g^{24}\\ g^{31} & g^{32} & 0 & g^{34}\\ g^{41} & g^{42} & g^{43} & 0 \end{array}\right).$$

Equation (2) is the general expression of the contravariant components of a space-time metric *g* in the basis of emission 1-forms $\left\{d\tau^{1},d\tau^{2},d\tau^{3},d\tau^{4}\right\}$. The vanishing of the diagonal terms characterizes the form of the contravariant gravitational potentials in the coordinate gauge imposed by the emission coordinates.

The emission coordinate three-surfaces are four one-parametric families of future null cones (with vertices on the word-line of the emitters) pairwise intersecting in six families of space-like coordinate two-surfaces, and three-wise intersecting in four families of space-like coordinate lines.

The Lorentzian character of the space-time, given by the signature (the signature of a metric is the sum of the signs of the terms of its diagonal form) sigg=−2ϵ, ϵ=±1, of its metric *g*, also imposes that all quantities $g^{AB}$ of the matrix (2) have the same sign *ϵ* (the covectors $d\tau^{A}$ are null and future-directed). It also imposes that the three geometric means $\sqrt{g^{12}g^{34}}$, $\sqrt{g^{13}g^{24}}$, and $\sqrt{g^{14}g^{23}}$ satisfy the inequalities of the sides of a triangle [24]. In this work we have chosen *ϵ* = −1.

The covariant potentials $g_{AB}$ of the metric *g* in this coordinate gauge are more complicated to obtain [24]. They are of the form $g_{AA}=\mu_{A}^{2}$, $g_{AB}=\mu_{A}\mu_{B}\cos\alpha_{AB}$, where the six angles $\alpha_{AB}$ are submitted to the four constraints $\alpha_{12}=\alpha_{34}$, $\alpha_{13}=\alpha_{24}$, $\alpha_{23}=\alpha_{14}$, and $\alpha_{12}+\alpha_{13}+\alpha_{23}=2\pi$ with $0<\alpha_{12},\alpha_{13},\alpha_{23}<\pi$.

Considered as physical coordinates, emission coordinates may seem striking at first, in part because they can never be at rest with respect to any observer, but also because they seem to lack the essential ingredients that are always present in classical coordinate systems. Nevertheless, according to [37], we must not forget that (i) to locate an object is to identify the place it takes up, (ii) consequently, to locate objects irrespective of their size we need to assign a proper name to every space-time event, (iii) since space-time is a four-dimensional continuum, assigning a proper name to its events has to be carried out with four numbers, and finally (iv) since a clock is a continuous generator of numbers, namely, the time that it displays at every instant, four clocks broadcasting their times by means of electromagnetic signals assign to every event four numbers and thus constitute a genuine coordinate system. Essentially, this initial hesitation is due to the prejudices induced by some familiar ingredients frequently present in many classical coordinate systems. Ingredients like origin, coordinate lines, or synchronization of a coordinate system are respectively irrelevant, of secondary interest, or inexistent in general coordinate systems and in RPSs in particular.

Emission coordinates constitute an interesting tool for the analysis of RPSs: as a field of continuous and differentiable data, and not only as simple parameters (pseudoranges) for specific real users that measure them, the usual techniques and methods of local differential geometry may be applied to their analysis, giving rise to simple and interesting physical interpretations (formally, considering the emission coordinates’ formalism as an alternative to the (discrete) pseudorange analysis is to consider the powerful local field formalism as an alternative to action-at-a-distance theories). But a RPS may consist of a constellation of more than four clocks. Then, what four satellites have to be chosen, among all of them, to construct the system of emission coordinates? In fact, if there are *k* > 4 satellites, depending on the world-lines of the clocks, we may be led to consider all the k4 emission coordinate systems generated by all the possible tetrads of clocks. They will constitute an atlas of local coordinate systems for the emission region R (whatever its topology) covered by these tetrads [38]. It is this atlas which defines precisely this region R. If R may be embedded in $R^{4}$, this atlas of local coordinate systems (charts) may be the starting point to define any ideal global coordinate system for R (for example, the ECR (Earth Centered Rotational) or the ECI (Earth Centered Inertial) reference frames). This is why RPSs may play the role of generators of primary coordinate systems [33]. In the next subsection, the gradients of the emission coordinates are analysed.

### 3.1. Emission 1-Forms $d\tau^{A}$

**Figure 1:**
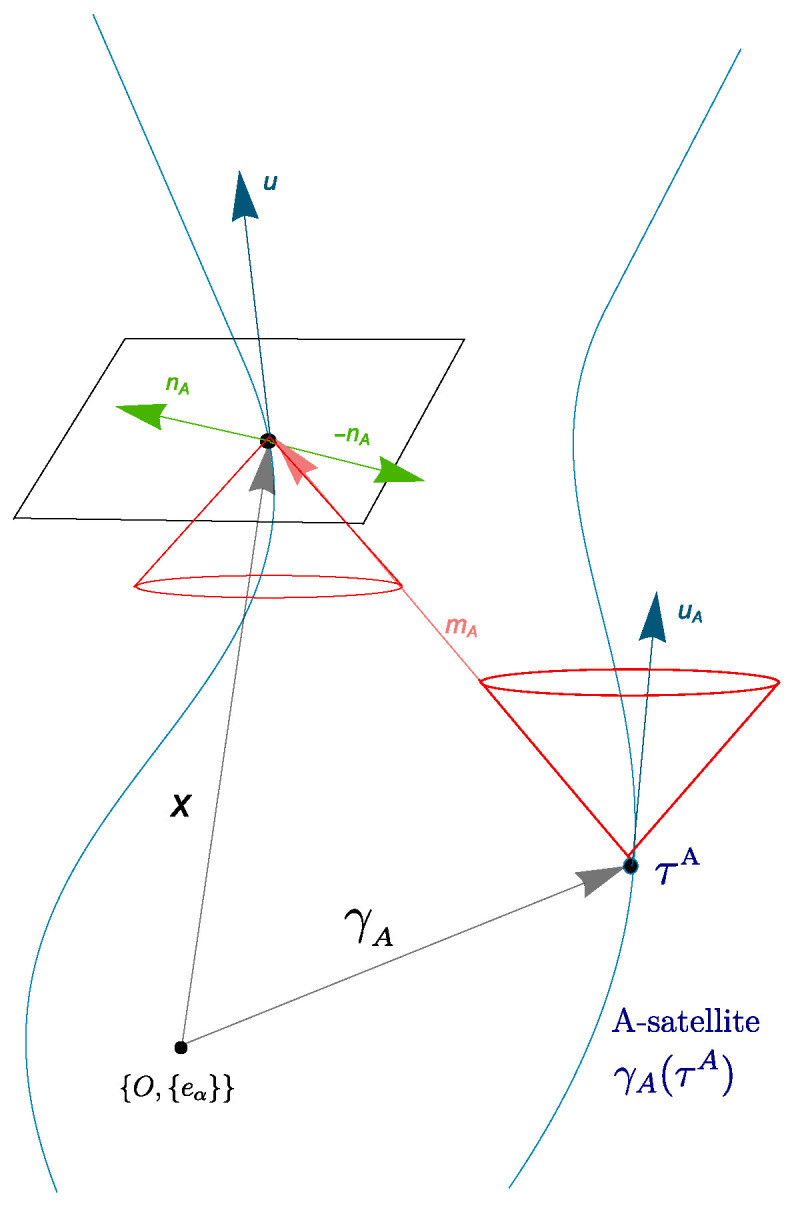
Configuration vectors in Minkowski space-time and unit vectors $n_{A}$ and $-n_{A}$ on the 3-space orthogonal to u.

Let x=γ(τ) be the world-line of the user of a given RPS and let $x^{\alpha }$ be the components of *x* in the inertial basis $\left\{e_{\alpha }\right\}$, $x=x^{\alpha }e_{\alpha }$. If $\left\{\tau^{A}\right\}$ are the emission coordinates of *x*, this means that the past light cone at event *x* intersects the emitters’ world-lines $\gamma_{A}$ at the emission events $\gamma_{A}\left(\tau^{A}\right)$, A=1,2,3,4. Consider the basis of 1-forms $\left\{d\tau^{A}\right\}$ defined by the emission coordinate differentials. The affine structure of Minkowski space-time allows us to define the vectors joining the reception event with the emission events,

(3) $$m_{A}=x-\gamma_{A},\;A=1,2,3,4,$$

which are null,

(4) $$0=m_{A}^{2}=g_{\alpha \beta }m_{A}^{\alpha }m_{A}^{\beta }=g_{\alpha \beta }\left(x^{\alpha }-\gamma_{A}^{\alpha }\right)\left(x^{\beta }-\gamma_{A}^{\beta }\right),$$

and future-oriented (see Figure 1). Taking the differential of (4) one obtains

(5) $$\begin{array}{ccc} 0 & = & 2g_{\alpha \beta }\left(dx^{\alpha }-{\dot{\gamma }}_{A}^{\alpha }\left(\tau^{A}\right)d\tau^{A}\right)\left(x^{\beta }-\gamma_{A}^{\beta }\left(\tau^{A}\right)\right)\\ & = & 2g_{\alpha \beta }\left[dx^{\alpha }-u_{A}^{\alpha }\left(\tau^{A}\right)\;d\tau^{A}\right]m_{A}^{\beta }=2\left[{\left(m_{A}\right)}_{\beta }\;dx^{\beta }-{\left(m_{A}\right)}_{\alpha }u_{A}^{\alpha }\;d\tau^{A}\right], \end{array}$$

with $u_{A}\equiv {\dot{\gamma }}_{A}\left(\tau^{A}\right)$ the unit velocity of emitter *A*, ${\left(u_{A}\right)}^{2}=-1$. Then,

(6) $${\underset{*}{m}}_{A}=\left(m_{A}\cdot u_{A}\right)\;d\tau^{A},\;d\tau^{A}=\frac{1}{m_{A}\cdot u_{A}}\;{\underset{*}{m}}_{A},$$

where a star under a letter is used to distinguish a covector, in this case ${\underset{*}{m}}_{A}$, from its metrically equivalent vector $m_{A}$.

On the other hand, if *u* is the unit velocity of the user, we have the relative decomposition

(7) $$u_{A}=w_{A}\left(u+\beta_{A}\right),\;u\cdot \beta_{A}=0,\;w_{A}={\left(1-\beta_{A}^{2}\right)}^{-1/2},$$

where $\beta_{A}$ is the relative velocity of emitter *A* (at the emission event) with respect to the user (at the reception event), and $w_{A}$ is the emitter–user relativistic factor.

Let us consider the time plus space decomposition of $m_{A}$ relative to *u*,

(8) $$m_{A}=-\left(m_{A}\cdot u\right)\left(u+n_{A}\right),\;u^{2}=-1,\;{\left(n_{A}\right)}^{2}=1,\;u\cdot n_{A}=0,$$

with $n_{A}$ being the unit vector along the direction of the signal broadcast by *A* with respect to the user *u* ($-n_{A}$ is the unit vector along the line of sight of the *A* emitter). Then, from (7),

(9) $$m_{A}\cdot u_{A}=-\left(m_{A}\cdot u\right)\;w_{A}\left(u+n_{A}\right)\cdot \left(u+\beta_{A}\right)=\left(m_{A}\cdot u\right)w_{A}\left(1-\beta_{A}\cdot n_{A}\right).$$

Let us consider the frequency ratio, $f_{A}$, between the received frequency by the user *u* and the corresponding one emitted by satellite *A*, which is expressed as

(10) $$f_{A}\equiv {\left[\frac{f_{received}}{f_{emitted}}\right]}_{A}=\frac{m_{A}\cdot u}{m_{A}\cdot u_{A}}=\frac{1}{w_{A}\left(1-\beta_{A}\cdot n_{A}\right)}.$$

For each emitter, $f_{A}$ is the ratio between the frequency received by the user and the proper frequency emitted by the *A* satellite. In GNSSs, the received signal is modelled as a linear superposition of the individual signals broadcast by the satellites in view, assigning an amplitude weight, a phase delay, and a frequency shift to each signal. For each satellite in view, the Doppler shift is given by $z_{A}=f_{A}-1$, with $f_{A}$ defined by Equation (10). Thus, the carrier frequency emitted by the *A* satellite (the central frequency of an emitted modulated signal) is identified with the emitted frequency $f_{emitted}$ and the corresponding shifted carrier frequency measured by the user with the received frequency $f_{received}$.

From (6), (8) and (10), the one-form of the *A* emission coordinate differential is written as

(11) $$d\tau^{A}=-f_{A}\;\left(\underset{*}{u}+{\underset{*}{n}}_{A}\right)=-\frac{\underset{*}{u}+{\underset{*}{n}}_{A}}{\omega_{A}\left(1-\beta_{A}\cdot n_{A}\right)}\;.$$

In the next section, we consider the future-oriented null vector $l_{A}$ defined by

(12) $$l_{A}=f_{A}\left(u+n_{A}\right),$$

which is (up to a sign) metrically equivalent to $d\tau^{A}$.

### 3.2. Jacobian Determinant of the Transformation from Inertial to Emission Coordinates and FGDOP

**Figure 2:**
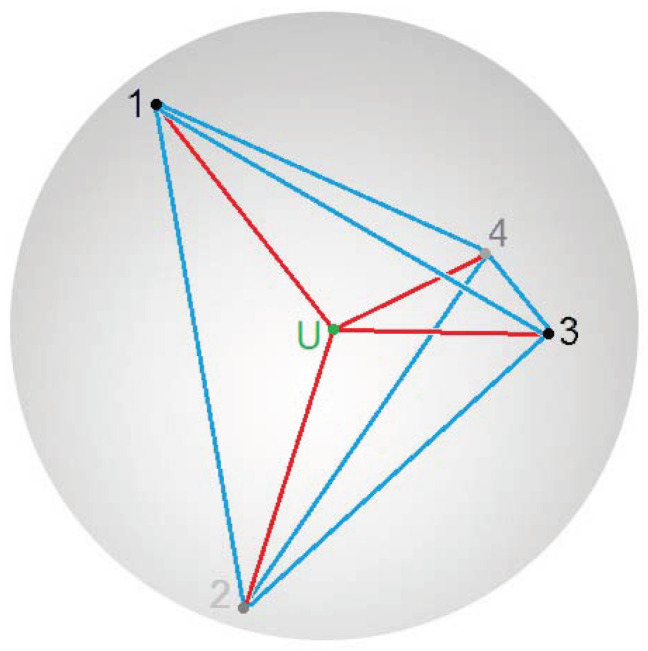
User unit celestial sphere $S_{U}$ and the inscribed tetrahedron defined by the relative positions of four emitters on it. Four inner tetrahedrons with common vertex on *U* and with a sole inscribed face on $S_{U}$ are also shown.

In this section and for *k* = 4, we show that the modulus of the Jacobian determinant J of the transformation $\tau^{A}\left(x^{\alpha }\right)$ from inertial to emission coordinates is given by |J|=6FV, where F is the product of the frequency ratios $f_{A}$ and V the volume of the inscribed tetrahedron that the four emitters define on the unit celestial sphere [51] of the user (see Figure 2).

Denote by *η* the metric volume element, $\eta =-\;\theta^{0}\wedge \theta^{1}\wedge \theta^{2}\wedge \theta^{3}$, with $\left\{\theta^{\alpha }\right\}$ the algebraic dual basis of the orthonormal basis $\left\{e_{\alpha }\right\}$,

(13) $$\eta_{\alpha \beta \gamma \delta }=-\sqrt{-\det g_{\lambda \rho }}\;\epsilon_{\alpha \beta \gamma \delta },\;\eta^{\alpha \beta \gamma \delta }=\frac{\epsilon^{\alpha \beta \gamma \delta }}{\sqrt{-\det g_{\lambda \rho }}},$$

where $\det g_{\lambda \rho }$ is the metric determinant in the coordinate system $\left\{x^{\alpha }\right\}$. Then we have

(14) $$\begin{array}{ccc} {}*(d\tau^{1}\wedge d\tau^{2}\wedge d\tau^{3}\wedge d\tau^{4}) & = & \eta_{\alpha \beta \gamma \delta }\;{\left(d\tau^{1}\right)}^{\alpha }\;{\left(d\tau^{2}\right)}^{\beta }\;{\left(d\tau^{3}\right)}^{\gamma }\;{\left(d\tau^{4}\right)}^{\delta }\\ & = & \eta^{\alpha \beta \gamma \delta }\;{\left(d\tau^{1}\right)}_{\alpha }\;{\left(d\tau^{2}\right)}_{\beta }\;{\left(d\tau^{3}\right)}_{\gamma }\;{\left(d\tau^{4}\right)}_{\delta }\\ & = & \frac{\epsilon^{\alpha \beta \gamma \delta }}{\sqrt{-\det g_{\lambda \rho }}}\;\frac{\partial \tau^{1}}{\partial x^{\alpha }}\;\frac{\partial \tau^{2}}{\partial x^{\beta }}\;\frac{\partial \tau^{3}}{\partial x^{\gamma }}\;\frac{\partial \tau^{4}}{\partial x^{\delta }}=\frac{J}{\sqrt{-\det g_{\lambda \rho }}}, \end{array}$$

where the star ∗ denotes the Hodge (or duality) operator acting on the space-time exterior algebra, and J is the Jacobian determinant of the transformation $\tau^{A}\left(x^{\alpha }\right)$.

From now on, we use the notation $\left\{x^{\alpha }\right\}\equiv \left\{x^{0},x^{1},x^{2},x^{3}\right\}$ for (Cartesian) inertial coordinates. Then, for the transformation from inertial to emission coordinates, $\tau^{A}\left(x^{\alpha }\right)$, the Jacobian determinant is given by

(15) $$J=*(d\tau^{1}\wedge d\tau^{2}\wedge d\tau^{3}\wedge d\tau^{4}).$$

The sign of J(x) is called the orientation of the RPS at the user event *x*. The determination of this orientation allows us to solve the bifurcation problem for Minkowskian RPSs [39], by distinguishing the space-time location of the user in inertial coordinates from another different one with the same emission coordinates. Then, substituting Equation (12) in (15), and denoting the product of the frequency ratios as $F\equiv \prod_{A=1}^{4}f_{A}$, we have the following:

(16) $$\begin{array}{ccc} J & = & *\left(l_{1}\wedge l_{2}\wedge l_{3}\wedge l_{4}\right)=F*\left[\left(u+n_{1}\right)\wedge \left(u+n_{2}\right)\wedge \left(u+n_{3}\right)\wedge \left(u+n_{4}\right)\right]\\ & = & F*\left[(u\wedge \left(n_{2}\wedge n_{3}\wedge n_{4}-n_{1}\wedge n_{3}\wedge n_{4}+n_{1}\wedge n_{2}\wedge n_{4}-n_{1}\wedge n_{2}\wedge n_{3}\right)\right]\\ & = & F\left[\left[i\left(u\right)\eta \right]\left(n_{2},n_{3},n_{4}\right)-\left[i\left(u\right)\eta \right]\left(n_{1},n_{3},n_{4}\right)+\left[i\left(u\right)\eta \right]\left(n_{1},n_{2},n_{4}\right)-\left[i\left(u\right)\eta \right]\left(n_{1},n_{2},n_{3}\right)\right]\\ & = & F\left[\left(n_{1},n_{2},n_{3}\right)+\left(n_{1},n_{3},n_{4}\right)-\left(n_{2},n_{3},n_{4}\right)-\left(n_{1},n_{2},n_{4}\right)\right]. \end{array}$$

For a given *p*-tensor *T*, the interior product i(u)T is the (p−1) tensor that results from the contraction of *u* with the first index of *T*. To reach the third and fourth equalities from the second one we have considered that

(17) $$\begin{array}{ccc} {}*(u\wedge n_{1}\wedge n_{2}\wedge n_{3}) & = & \left[i\left(u\right)\;\eta \right]\left(n_{1},n_{2},n_{3}\right)\; \end{array}$$

(18) $$\begin{array}{ccc} \; & = & -\eta_{3}\left(n_{1},n_{2},n_{3}\right)\equiv -\left(n_{1},n_{2},n_{3}\right), \end{array}$$

similarly for all the involved summands, where, in (17), $\left(n_{1},n_{2},n_{3}\right)$ is the argument of the trilinear form [i(u)η] and, in (18), $\eta_{3}\equiv -i\left(u\right)\;\eta$ is the 3-volume metric element relative to *u* and $\left(x,y,z\right)\equiv \;\eta_{3}\left(x,y,z\right)$ denotes the mixed product on the Euclidean space orthogonal to *u*, that is, $\left(x,y,z\right)=\left(\theta^{1}\wedge \theta^{2}\wedge \theta^{3}\right)\left(x,y,z\right)=\varepsilon_{abc}x^{a}y^{b}z^{c}.$

With the notation

(19) $$\mu_{a}\equiv n_{a}-n_{4},\;\;a=1,2,3,$$

the expression (16) for the Jacobian determinant and Equation (10) for $f_{A}$ lead to the following result.

**Proposition 1**  (Jacobian determinant of $\left\{\tau^{A}\left(x^{\alpha }\right)\right\}$)**.** *The Jacobian determinant, J, of the inertial to emission coordinate transformation $\tau^{A}\left(x^{\alpha }\right)$ is given by the following:* 

(20) $$J\left(x\right)=\;\left(\mu_{1},\mu_{2},\mu_{3}\right)\;F,\;F=\prod_{A=1}^{4}f_{A},\;\;f_{A}=\frac{\sqrt{1-{\left(\beta_{A}\right)}^{2}}}{1-\beta_{A}\cdot n_{A}},$$

*where $f_{A}$ stands for the frequency ratio of the emitter $\gamma_{A}$, with $n_{A}$ the unit vector along the direction of propagation of the signal broadcast by emitter A, as seen by the user, and $\mu_{a}=n_{a}-n_{4}$, a=1,2,3.*

For non-relativistic velocities at first order approximation,

(21) $$F=1+\sum_{A=1}^{4}\beta_{A}\cdot n_{A}.$$

The volume V of the tetrahedron defined for a quad of emitters on the unit celestial sphere of the user is given by

(22) $$V=\frac{1}{6}\;\left|\left(\mu_{1},\mu_{2},\mu_{3}\right)\right|=\frac{\left|J\right|}{6F}\;,$$

and, for a static situation, $\beta_{A}=0$, F=1, and |J|=6V. For a regular tetrahedron inscribed on a unit 2-sphere, $V_{reg}=\frac{8}{9\sqrt{3}}$, the Jacobian reaches the maximum, $J_{max}=\frac{16}{3\sqrt{3}}\approx 3.08$. In this case, the angle between each pair of vertices is $\alpha =\arccos\left(-\frac{1}{3}\right)=109{}^{\circ}\;28^{\prime }\;16.39^{\prime \prime }$, and the dihedral angle $\theta =\pi -\alpha =\arccos\left(\frac{1}{3}\right)=70{}^{\circ}\;31^{\prime }\;43.61^{\prime \prime }$.

From the geometric interpretation provided by Proposition 10 in Section 6 and Equation (22), it results that for a RPS constituted by four emitters the *FGDOP* scalar is inversely proportional to the Jacobian determinant, J, of the transformation from inertial to emission coordinates.

## 4. The Frequency Geometric Matrix

In classical positioning, a geometric 4 × 4 matrix $G_{c}$ is associated with the positions of the emitters on the sky. The GDOP matrix is the inverse of this geometric matrix, $G_{c}^{-1}$ ($G_{c}$ is supposedly regular). In this section, this notion will be extended to a relativistic concept by including the frequency ratios and making it appropriate for RPS theory.

### 4.1. The Geometric Matrix $G_{c}$ of a Set of k Emitters

We start by introducing the classical geometric matrix $G_{c}$ for any number *k* of emitter points. Using the language of Global Navigation Satellite Systems (GNSSs), let us consider a user instantaneously placed at a point *P* and a set of 3-dimensional unit vectors $\left\{n_{1},\ldots ,n_{k}\right\}$. In this work $-n_{A}$ is the unit vector along the line of sight at *P*, from the receiver (user) to emitter *A* (satellite), for each A=1,2,…,k. Let $H_{c}$ be the *k* × 4 matrix,

(23) $$H_{c}=\left(\begin{array}{cccc} 1 & n_{1_{x}} & n_{1_{y}} & n_{1_{z}}\\ 1 & n_{2_{x}} & n_{2_{y}} & n_{2_{z}}\\ \ldots & \ldots & \ldots & \ldots \\ 1 & n_{k_{x}} & n_{k_{y}} & n_{k_{z}} \end{array}\right)$$

defined from the Cartesian components of the unit vectors $n_{A}=\left(n_{A_{x}},n_{A_{y}},n_{A_{z}}\right)$, A=1,…,k, and adding a first column made of *k* ones. Different terms are used to refer to the matrix $H_{c}$ (design matrix in [2], visibility matrix in [52], design or figure matrix in [50]). The matrix $H_{c}$ follows from linearizing the Euclidean distances between the satellites (at emission time) and the receiver (at reception time) and allows us to write the linearized navigation equations in matrix form [1,2,3]. In the GNSS, the column of ones is interpreted as the derivative of the pseudoranges with respect the user clock bias (note that *c* = 1).

The convention followed in (23) is consistent with the order of Minkowskian inertial coordinates $\left\{x^{0}=ct,x^{1}=x,x^{2}=y,x^{3}=z\right\}$ used in the manuscript, where the first coordinate is the coordinate inertial time (in length units). In other words, $H_{c}$ is the matrix formed by the coefficients of the linearized navigation equations by considering clock offset and user position coordinates as unknowns to be determined.

Apart from the position of the column of ones, note that the classical design matrix found in the literature differs from $H_{c}$ (23) by a sign. This difference obeys the meaning of $-n_{A}$ as the unit direction along the line of sight of the *A* emitter. Nevertheless, the sign differences are not relevant: neither for the geometric classical product matrix $G_{c}=H_{c}^{T}\times H_{c}$ (24), nor for the Gram matrix elements that involve the Euclidean scalar products $n_{A}\cdot n_{B}=\cos\theta_{AB}$.

Then, if $H_{c}^{T}$ denotes the transpose of $H_{c}$, the matrix $G_{c}$ is defined as the 4 × 4 matrix given by the product $H_{c}^{T}\times H_{c}$, that is,

(24) $$G_{c}\equiv H_{c}^{T}\times H_{c}=\left(\begin{array}{cccc} k & \sum_{A=1}^{k}n_{A_{x}} & \sum_{A=1}^{k}n_{A_{y}} & \sum_{A=1}^{k}n_{A_{z}}\\ \sum_{A=1}^{k}n_{A_{x}} & \sum_{A=1}^{k}{\left(n_{A_{x}}\right)}^{2} & \sum_{A=1}^{k}n_{A_{x}}n_{A_{y}} & \sum_{A=1}^{k}n_{A_{x}}n_{A_{z}}\\ \sum_{A=1}^{k}n_{A_{y}} & \sum_{A=1}^{k}n_{A_{x}}n_{A_{y}} & \sum_{A=1}^{k}{\left(n_{A_{y}}\right)}^{2} & \sum_{A=1}^{k}n_{A_{y}}n_{A_{z}}\\ \sum_{A=1}^{k}n_{A_{z}} & \sum_{A=1}^{k}n_{A_{x}}n_{A_{z}} & \sum_{A=1}^{k}n_{A_{y}}n_{A_{z}} & \sum_{A=1}^{k}{\left(n_{A_{z}}\right)}^{2} \end{array}\right),$$

which may be in short expressed as

(25) $$G_{c}=\sum_{A=1}^{k}\left(\begin{array}{cccc} 1 & n_{A_{x}} & n_{A_{y}} & n_{A_{z}}\\ n_{A_{x}} & {\left(n_{A_{x}}\right)}^{2} & n_{A_{x}}n_{A_{y}} & n_{A_{x}}n_{A_{z}}\\ n_{A_{y}} & n_{A_{x}}n_{A_{y}} & {\left(n_{A_{y}}\right)}^{2} & n_{A_{y}}n_{A_{z}}\\ n_{A_{z}} & n_{A_{x}}n_{A_{z}} & n_{A_{y}}n_{A_{z}} & {\left(n_{A_{z}}\right)}^{2} \end{array}\right).$$

In the following subsection, by including the frequency ratios as a factor in the null vectors $l_{A}$, the classical geometric matrix is generalized in the framework of RPS theory.

### 4.2. The Frequency Geometric Matrix G of a Set of k Emitters

In a relativistic context, the essential elements in the definition of $H_{c}$ and $G_{c}$ are null vectors conveniently parametrized with respect to an observer. Given a space-time observer *u*, let us consider the null vectors defined by (12)

(26) $$l_{A}=f_{A}\left(u+n_{A}\right),\;A=1,\ldots ,k,$$

where $f_{A}=-u\cdot l_{A}$ is the frequency ratio measured by *u*; the vector $n_{A}$ (being unitary $n_{A}^{2}=1$ and orthogonal to *u*) gives the relative direction of propagation of the signal coming from emitter *A*. Then, the components of $l_{A}$ in an orthochronous frame adapted to *u* lead to the definition of the Frequency-Visibility matrix *H* as

(27) $$H=\left(\begin{array}{cccc} f_{1} & f_{1}n_{1_{x}} & f_{1}n_{1_{y}} & f_{1}n_{1_{z}}\\ f_{2} & f_{2}n_{2_{x}} & f_{2}n_{2_{y}} & f_{2}n_{2_{z}}\\ \ldots & \ldots & \ldots & \ldots \\ f_{k} & f_{k}n_{k_{x}} & f_{k}n_{k_{y}} & f_{k}n_{k_{z}} \end{array}\right)$$

and the Frequency Geometric matrix $G\equiv H^{T}\times H$ as

(28) $$G=\sum_{A=1}^{k}{\left(f_{A}\right)}^{2}\;C_{A},\;\;C_{A}\equiv \left(\begin{array}{cccc} 1 & n_{A_{x}} & n_{A_{y}} & n_{A_{z}}\\ n_{A_{x}} & {\left(n_{A_{x}}\right)}^{2} & n_{A_{x}}n_{A_{y}} & n_{A_{x}}n_{A_{z}}\\ n_{A_{y}} & n_{A_{x}}n_{A_{y}} & {\left(n_{A_{y}}\right)}^{2} & n_{A_{y}}n_{A_{z}}\\ n_{A_{z}} & n_{A_{x}}n_{A_{z}} & n_{A_{y}}n_{A_{z}} & {\left(n_{A_{z}}\right)}^{2} \end{array}\right).$$

If $f_{A}=1$ for all emitters, $G=G_{c}$. However, to obtain the relativistic generalization of classical GDOP concepts for non-static situations, it is essential to include the measured frequencies in the definition of *G*.

Note that the Frequency Geometric Matrix can be written as follows:

(29) $$G=H_{c}^{T}\times F^{2}\times H_{c},$$

where *F* is the diagonal matrix of frequency ratios:

(30) $$F=\left(\begin{array}{ccc} f_{1} & \; & 0\\ \; & \ddots & \;\\ 0 & \; & f_{k} \end{array}\right).$$

Formally, the Frequency Geometric Matrix *G* is equivalent to a weighted GDOP matrix (see [19]), with *F* acting as a weight matrix and the frequency ratios $f_{A}$ as the inverse of the covariances $\sigma_{A}$ of measurement errors.

At each space-time event (ct,x,y,z), the set of differential 1-forms ${\left\{d\tau^{A}\right\}}_{A=1}^{k}$ is a linear map that transforms the vector increment (cΔt,Δx,Δy,Δz) into the *k*-dimensional vector increment $\left(\Delta \tau^{1},\Delta \tau^{2},\ldots ,\Delta \tau^{k}\right)$ of the emission coordinate grid space $\left\{\left(\tau^{1},\tau^{2},\ldots ,\tau^{k}\right)\right\}$ defined by the ordered sets of satellite proper time values. This is the interpretation of Equation (11) for positioning error analysis including Doppler effects in the $f_{A}$ frequency ratios. The effective result is similar to the one obtained from a weighted pseudorange positioning error method (with weights accounting for Doppler effects). For uniform (respectively, non-uniform) Doppler error distributions, the information is encoded in frequency ratios that are all equal (resp., different). For example, this is the case for a RPS consisting of a regular tetrahedral configuration, where each satellite moves along the symmetry axis passing through a vertex, with equal (resp., different) constant velocities, and for the FGDOP scalar obtained at the centre of the configuration.

### 4.3. The Contravariant 2-Tensor L Associated to k Null Vectors

Given a set of *k* null vectors, it is apparent that the Frequency Geometric Matrix *G* and the tensor defined as

(31) $$L=\sum_{A=1}^{k}l_{A}⊗l_{A}$$

are closely related. In fact, if $\left\{e_{0},e_{1},e_{2},e_{3}\right\}$ is an orthonormal basis with $e_{0}=u$, we have $L=L^{\mu \nu }e_{\mu }⊗e_{\nu }$. The components $L^{\mu \nu }$ of *L* in this basis are the entries of the geometric matrix $G_{\;\;\nu }^{\mu }$ given by (28). However, (31) is not the adequate covariantization of *G*; this is accomplished in Section 4.4 below.

Given an observer *u*, the symmetric 2-tensor *L* admits the 3 + 1 decomposition:

(32) $$L=\sum_{A=1}^{k}l_{A}⊗l_{A}=\sum_{A=1}^{k}f_{A}^{2}\left(u+n_{A}\right)⊗\left(u+n_{A}\right)=K\;u⊗u+u\tilde{⊗}e+E$$

where $K\equiv \sum_{A=1}^{k}f_{A}^{2}$, $u\tilde{⊗}e\equiv u⊗e+e⊗u$ stands for the symmetrized tensor product and *e* and *E* are given by

(33) $$e\equiv \sum_{A=1}^{k}f_{A}^{2}\;n_{A},\;E\equiv \sum_{A=1}^{k}f_{A}^{2}\;n_{A}⊗n_{A},$$

and are called, respectively, the frequency-weighted vector axis and the frequency-weighted tensor axis of *L* relative to *u*, both axes belonging to the 3-space orthogonal to *u*. Notice that the trace of the tensor axis is equal to *K*,

(34) $$\mathrm{tr}E=\sum_{A=1}^{k}f_{A}^{2}\;n_{A}\cdot n_{A}=\sum_{A=1}^{k}1_{A}f_{A}^{2}=K,$$

where $1_{A}\equiv 1$ (for all A=1,2,…,k) has been included to make the summation explicit. Thus, if N is the k×k Gram matrix of the set of vectors ${\left\{f_{A}n_{A}\right\}}_{A=1}^{k}$, whose entries are $N_{AB}=f_{A}f_{B}n_{A}\cdot n_{B}$, then trE=trN. Moreover, $E^{p}$ and $N^{p}$ have equal trace,

(35) $$\mathrm{tr}E^{p}=\mathrm{tr}N^{p}$$

for every integer *p*.

When the frequency ratios are all equal to one, $f_{A}=1_{A}$, the algebraic structure of *E* (or, equivalently, that of N) is strongly connected with the symmetric configurations of emitters on the user’s unit celestial sphere (see Section 7).

### 4.4. Tensor Expression for G

The Frequency Geometric Matrix *G* (28) is a relative notion because it involves a given, otherwise arbitrary, space-time observer *u*. Nevertheless, it makes sense (i) to look for a covariant expression for *G* in which the dependence on *u* becomes more explicit, and then (ii) to ask for the transformation of *G* under a change of observer (orthochronous Lorentz transformation). To this end, as a matter of convenience, let us introduce the subsidiary positive definite metric tensor $g_{u}$, defined from the space-time metric *g* and the observer *u* by the relation

(36) $$g_{u}=g+2\underset{*}{u}⊗\underset{*}{u}$$

where $\underset{*}{u}$ is the covector metrically equivalent to *u* (its covariant components are obtained by lowering the contravariant index of *u* with the metric, ${\left(\underset{*}{u}\right)}_{\mu }\equiv u_{\mu }=g_{\mu \nu }u^{\nu }$).

The entries of the geometric matrix are the components, in an orthonormal basis adapted to *u*, of the mixed tensor

(37) $$G=L\times g_{u}\;,$$

where, as in (24), × stands for the usual matrix product (contraction of adjacent indexes). We develop this matrix product by using tensor components

$$\begin{array}{ccc} G_{\;\;\nu }^{\mu } & = & {\left(L\times g_{u}\right)}_{\;\;\nu }^{\mu }=L^{\mu \rho }{\left(g_{u}\right)}_{\rho \nu }=L^{\mu \rho }\left(g_{\rho \nu }+2u_{\rho }u_{\nu }\right)=L_{\;\;\nu }^{\mu }+2L^{\mu \rho }u_{\rho }u_{\nu }\\ & = & L_{\;\;\nu }^{\mu }+2\sum_{A=1}^{k}{\left(l_{A}\right)}^{\mu }{\left(l_{A}\right)}^{\rho }u_{\rho }u_{\nu }=L_{\;\;\nu }^{\mu }-2\sum_{A=1}^{k}f_{A}{\left(l_{A}\right)}^{\mu }u_{\nu }, \end{array}$$

where, in the last step, we have taken into account that $l_{A}\cdot u=-f_{A}$ for any emitter. Using index free tensor notation, the following result is obtained.

**Proposition 2**  (Tensor expression for the Frequency Geometric Matrix *G*)**.** *Given an observer u, the frequency geometric tensor G associated with k null vectors ${\left\{l^{A}\right\}}_{A=1}^{k}$, is expressed as*

(38) $$G=\sum_{A=1}^{k}l_{A}⊗\;{\underset{*}{l}}_{A}-2\left(\sum_{B=1}^{k}f_{B}l_{B}\right)⊗\;\underset{*}{u}$$

where $f_{A}=-u\cdot l_{A}$, and the star under a letter is used to distinguish a covector, say $\underset{*}{v}$, from its metrically equivalent vector v.

Moreover, substituting (26) into (38), we have the following decomposition of *G*,

(39) $$G=Ku⊗\underset{*}{u}+u⊗\underset{*}{e}+e⊗\underset{*}{u}+E-2\left(Ku+e\right)⊗\underset{*}{u}=-Ku⊗\underset{*}{u}+u⊗\underset{*}{e}-e⊗\underset{*}{u}+E$$

with *e* the frequency-weighted vector axis and $E\equiv \sum_{A=1}^{k}f_{A}^{2}\;n_{A}⊗\underset{*}{n_{A}}$, the mixed frequency-weighted tensor axis.

## 5. The Gram Matrix, G, of k Null Vectors

Let us denote by $G\equiv G_{k\times k}$ the Gram matrix of an ordered set of *k* future-oriented null vectors ${\left\{l_{A}\right\}}_{A=1}^{k}$. By definition, G is the k×k matrix whose entries, $G_{AB}$, are the scalar products of the given vectors with the space-time metric *g*, that is,

(40) $$G_{AB}\equiv g\left(l_{A},l_{B}\right)\equiv l_{A}\cdot l_{B},\;A,B=1,\ldots ,k.$$

Consequently, G is symmetric, its diagonal entries vanish ($G_{AA}=l_{A}^{2}=0$) and, for k>4, detG = 0. Taking into account (26), we have

(41) $$G_{AB}=f_{A}f_{B}\left(-1+\cos\theta_{AB}\right),$$

since $u^{2}=-1$ and $u\cdot n_{A}=u\cdot n_{B}=0$, and where $\theta_{AB}\in \left[0,\pi \right]$ is the angle between emitters *A* and *B* as measured by observer *u*, that is, $c_{AB}\equiv \cos\theta_{AB}=n_{A}\cdot n_{B}$.

At a given space-time event, the set of unit vectors orthogonal to *u* defines the observer’s unit celestial sphere at this event [51]. We shall call this unit celestial sphere $S_{U}$. Then, If $d_{AB}$ is the length of the Euclidean segment defined by the relative positions of emitters *A* and *B* on $S_{U}$,

(42) $$d_{AB}^{2}={\left(n_{A}-n_{B}\right)}^{2}=2\left(1-\cos\theta_{AB}\right)=4\sin^{2}\frac{\theta_{AB}}{2}=-2\frac{G_{AB}}{f_{A}f_{B}},$$

that is,

(43) $$d_{AB}=2\sin\frac{\theta_{AB}}{2},$$

and the area of the triangle of vertices UAB is given by $\frac{1}{2}\sin\theta_{AB}$.

Incidentally, notice that, in terms of the frequency visibility matrix *H* and its transpose $H^{T}$, the Gram matrix can be expressed as a product of these matrices which also involves the space-time metric *g*,

(44) $$G=H\times g\times H^{T}.$$

Equation (44) is easily verified in an orthonormal basis ${\left\{e_{\alpha }\right\}}_{\alpha =0}^{3}$ adapted to the user, $u\equiv e_{0}$, with $g_{\alpha \beta }=g\left(e_{\alpha },e_{\beta }\right)=diag\left(-1,1,1,1\right)\equiv \eta_{\alpha \beta }$ and *H* and $H^{T}$ given by (23) and its transpose, respectively. Once verified in a particular matrix representation attached to an orthonormal space-time basis (or Cartesian inertial coordinate system), this expression remains valid in any space-time basis (or coordinate system).

### 5.1. Gram Principal Minors: Geometric Interpretation

For *k* ≥ 4, the only non-vanishing principal minors of G are those of second, third and fourth order, which are given by the following:

(45) $$\Delta_{AB}\equiv \left|\begin{array}{cc} 0 & G_{AB}\\ G_{AB} & 0 \end{array}\right|=-{\left(G_{AB}\right)}^{2},$$

(46) $$\Delta_{ABC}\equiv \left|\begin{array}{ccc} 0 & G_{AB} & G_{AC}\\ G_{AB} & 0 & G_{BC}\\ G_{AC} & G_{BC} & 0 \end{array}\right|=2G_{AB}G_{AC}G_{BC}=2G_{AB}G_{BC}G_{CA},$$

and

(47) $$\begin{array}{ccc} \Delta_{ABCD} & \equiv & \left|\begin{array}{cccc} 0 & G_{AB} & G_{AC} & G_{AD}\\ G_{AB} & 0 & G_{BC} & G_{BD}\\ G_{AC} & G_{BC} & 0 & G_{CD}\\ G_{AD} & G_{BD} & G_{CD} & 0 \end{array}\right|\\ & = & {\left(G_{AB}\right)}^{2}{\left(G_{CD}\right)}^{2}+{\left(G_{AC}\right)}^{2}{\left(G_{BD}\right)}^{2}+{\left(G_{AD}\right)}^{2}{\left(G_{BC}\right)}^{2}\\ & & -2(G_{AB}G_{AC}G_{BD}G_{CD}+G_{AB}G_{AD}G_{BC}G_{CD}+G_{AC}G_{AD}G_{BC}G_{BD}), \end{array}$$

respectively. These minors are invariant under the permutation of the involved null vectors and are related with dimensionless Euclidean lengths, areas, and volumes defined by the relative positions of the emitters on $S_{U}$, according to the following properties.

**Proposition 3**  (Geometric interpretation of the Gram principal minors, $\Delta_{AB}$, $\Delta_{ABC}$, and $\Delta_{ABCD}$)**.**

*(i)*  *Let $d_{AB}$ be the dimensionless length of the rectilinear segment defined by the relative positions of a pair of emitters {A,B} on $S_{U}$; then,*
  (48) $$\Delta_{AB}=-\frac{1}{4}{\left(f_{A}f_{B}d_{AB}^{2}\right)}^{2}$$
*(ii)*  *Let $A_{ABC}$ be the dimensionless area of the triangle defined by the relative positions of a triad of emitters {A,B,C} on $S_{U}$, and let $V_{UABC}$ be the dimensionless volume of the tetrahedron defined by the vertices of this triangle and the user position U (located at the centre of $S_{U}$); then,*
  (49) $$\Delta_{ABC}={\left(f_{A}f_{B}f_{C}\right)}^{2}\;\left[{\left(6V_{UABC}\right)}^{2}-{\left(2A_{ABC}\right)}^{2}\right].$$
*(iii)*  *Let $V_{ABCD}$ be the dimensionless volume of the* inscribed *tetrahedron defined by a quad of emitters {A,B,C,D} on $S_{U}$; then,*
  (50) $$\Delta_{ABCD}=-{\left(6f_{A}f_{B}f_{C}f_{D}V_{ABCD}\right)}^{2}.$$

*The frequency ratios $f_{A}$ are given by (10).*

All distances, areas, and volumes considered are dimensionless because they are associated to dimensionless unit vectors. Figure 2 shows a sole quad of emitters, numbered from 1 to 4, on $S_{U}$.

The proof of Proposition 3 is presented in Appendix A.

### 5.2. Traces of the Powers of G

Up to fourth order, the traces of the successive powers of G are as follows:

(51) $$\begin{array}{cc} \mathrm{tr}G\; & =0,\; \end{array}$$

(52) $$\begin{array}{cc} \;\;\mathrm{tr}G^{2} & =\sum_{A,B=1}^{k}G_{AB}G_{BA}=\sum_{A,B=1}^{k}{\left(G_{AB}\right)}^{2}=2\sum_{A<B}^{k}{\left(G_{AB}\right)}^{2}=-2\sum_{A<B}^{k}\Delta_{AB}, \end{array}$$

(53) $$\begin{array}{cc} \mathrm{tr}G^{3} & =\sum_{A,B,C=1}^{k}G_{AB}G_{BC}G_{CA}=6\sum_{A<B<C}^{k}G_{AB}G_{BC}G_{CA}=3\sum_{A<B<C}^{k}\Delta_{ABC}, \end{array}$$

(54) $$\begin{array}{cc} \mathrm{tr}G^{4} & =\sum_{A,B,C,D=1}^{k}G_{AB}G_{BC}G_{CD}G_{DA}=\sum_{A,B=1}^{k}{\left[{\left(G^{2}\right)}_{AB}\right]}^{2}.\; \end{array}$$

The third equality of (52) follows from $G_{AA}=0$ and $G_{AB}=G_{BA}$, since there are only k2=k(k−1)2! different non-vanishing summands; the fourth equality follows from (45). Similarly, the second equality of (53) follows since there are only k3=k(k−1)(k−2)3! different non-vanishing summands; the third equality follows from (46). From now on, we introduce the following notation for these traces,

(55) $$\sigma \equiv \mathrm{tr}G^{2},\;\lambda \equiv \mathrm{tr}G^{3},\;\omega \equiv \mathrm{tr}G^{4}.$$

Notice that the trace of the fifth and higher powers of G do not vanish. Nevertheless, these traces are not relevant for the present study and are not considered here.

In addition, we will denote the following:

(56) $$\begin{array}{ccc} S & \equiv & \sum_{A,B=1}^{k}f_{A}f_{B}G_{AB}=2\sum_{A<B}^{k}f_{A}f_{B}G_{AB}\; \end{array}$$

(57) $$\begin{array}{ccc} \Sigma & \equiv & \sum_{A,B,C=1}^{k}f_{A}f_{C}G_{AB}G_{BC}=\sum_{A,B=1}^{k}f_{A}f_{B}{\left(G^{2}\right)}_{AB}\; \end{array}$$

(58) $$\begin{array}{ccc} \Lambda & \equiv & \sum_{A,B,C,D=1}^{k}f_{A}f_{D}G_{AB}G_{BC}G_{CD}=\sum_{A,B=1}^{k}f_{A}f_{B}{\left(G^{3}\right)}_{AB}. \end{array}$$

The reason for this notation will become apparent in Section 5, where an expression of the *FGDOP* scalar in terms of quantities *K*, *σ*, *λ*, *S*, Σ, and Λ is given for a regular Frequency Geometric Matrix (detG≠0) of *k* ≥ 4 emitters (see Proposition 9). The quantity $\omega =\mathrm{tr}G^{4}$ is not explicitly needed since it can be obtained from *K*, *S*, *σ*, *λ*, and Λ as a consequence of the angular-Doppler identities between pairs of emitters (see Proposition 5). But, before we accomplish that, we summarize the following geometric relations between the quantities involved.

**Proposition 4**  (Doppler-weighted sums of segment lengths, triangle areas, and tetrahedron volumes)**.** *Rectilinear segment lengths $d_{AB}$, triangle areas $A_{ABC}$, and tetrahedron volumes $V_{ABCD}$ defined by the emitters’ relative positions on $S_{U}$, quantities s, σ, λ, *Σ*, and *Λ* defined above, the frequency ratios $f_{A}$ and $K=\sum_{A=1}^{k}f_{A}^{2}$ satisfy the following:*

*(i)*  *Doppler-weighted sum of the fourth power of the rectilinear lengths on $S_{U}$,*
  (59) $$\sum_{A<B}^{k}{\left(f_{A}f_{B}d_{AB}^{2}\right)}^{2}=2\sigma$$
*(ii)*  *Doppler-weighted sum of squared triangle areas on $S_{U}$,*
  (60) $$\sum_{A<B<C}^{k}{\left(2f_{A}f_{B}f_{C}A_{ABC}\right)}^{2}=\Sigma -K\frac{\sigma }{2}.$$
*(iii)*  *Doppler-weighted sum of squared tetrahedron volumes on $S_{U}$,*
  (61) $$\sum_{A<B<C<D}^{k}{\left(6f_{A}f_{B}f_{C}f_{D}V_{ABCD}\right)}^{2}=-\Lambda +K\frac{\lambda }{3}+S\frac{\sigma }{2}.$$

The proof of this proposition is presented in Appendix B.

### 5.3. Gram Characteristic Equation

From the Cayley–Hamilton theorem [53], the Gram matrix G of *k* ≥ 4 null vectors satisfies the characteristic matrix equation:

(62) $$G^{k-4}\left[G^{4}-\frac{\sigma }{2}G^{2}-\frac{\lambda }{3}G+\frac{1}{4}\left(\frac{\sigma^{2}}{2}-\omega \right)I\right]=0$$

where I is the k×k unit matrix. The coefficients of this equation are obtained from the traces of the powers of G (given by (55)), which are related to the sum of the principal minors of the Gram matrix by the following expressions:

(63) $$\begin{array}{cc} \;\frac{\sigma }{2} & =-\sum_{A<B}^{k}\Delta_{AB}=\frac{1}{4}\sum_{A<B}^{k}{\left(f_{A}f_{B}d_{AB}^{2}\right)}^{2},\; \end{array}$$

(64) $$\begin{array}{cc} \;\frac{\lambda }{3} & =\sum_{A<B<C}^{k}\Delta_{ABC}=\sum_{A<B<C}^{k}{\left(f_{A}f_{B}f_{C}\right)}^{2}\left[{\left(6V_{UABC}\right)}^{2}-{\left(2A_{ABC}\right)}^{2}\right], \end{array}$$

(65) $$\begin{array}{cc} \frac{1}{4}\left(\frac{\sigma^{2}}{2}-\omega \right) & =\sum_{A<B<C<D}^{k}\Delta_{ABCD}=-\sum_{A<B<C<D}^{k}{\left(6f_{A}f_{B}f_{C}f_{D}V_{ABCD}\right)}^{2}.\; \end{array}$$

The first equalities in (63)–(65) are well known from elemental algebra. They are directly verified by taking sums in (45)–(47)

(66) $$\begin{array}{cc} \sum_{A,B=1}^{k}\Delta_{AB} & =-\sum_{A,B=1}^{k}{\left(G_{AB}\right)}^{2}=-\sum_{A,B=1}^{k}G_{AB}G_{BA}=-\mathrm{tr}\;G^{2},\; \end{array}$$

(67) $$\begin{array}{cc} \sum_{A,B,C=1}^{k}\Delta_{ABC} & =2\sum_{A,B,C=1}^{k}G_{AB}G_{BC}G_{CA}=2\mathrm{tr}G^{3},\; \end{array}$$

(68) $$\begin{array}{cc} \sum_{A,B,C,D=1}^{k}\Delta_{ABCD} & =3\sum_{A,B=1}^{k}{\left(G_{AB}\right)}^{2}\sum_{C,D}^{k}{\left(G_{CD}\right)}^{2}-6\sum_{A,B,C,D=1}^{k}G_{AB}G_{BC}G_{CD}G_{DA}\\ & =3{\left(\mathrm{tr}G^{2}\right)}^{2}-6\mathrm{tr}G^{4}, \end{array}$$

and subsequently dividing by 2!, 3!, and 4!, respectively. From (61) and (65), the following result is obtained.

**Proposition 5**  (Doppler-weighted sum of the inscribed tetrahedrons squared volumes)**.** *The following relations hold:*

(69) $$\sum_{A<B<C<D}^{k}{\left(6f_{A}f_{B}f_{C}f_{D}V_{ABCD}\right)}^{2}=\frac{1}{4}(\omega -\frac{\sigma^{2}}{2})=K\;\frac{\lambda }{3}+S\;\frac{\sigma }{2}-\Lambda ,$$

*where $\omega \equiv \mathrm{tr}G^{4}$, $\lambda \equiv \mathrm{tr}G^{3}$, $\sigma \equiv \mathrm{tr}G^{2}$, and $\Lambda \equiv \sum_{A,B=1}^{k}f_{A}f_{B}{\left(G^{3}\right)}_{AB}$ are computed from the elements of the Gram matrix G which are given by the angular separation between emitters $\theta_{AB}$ and the frequency ratios $f_{A}$, that is, $G_{AB}=f_{A}f_{B}\left(-1+\cos\theta_{AB}\right)$.*

Notice that the left-hand side of the second equality, which is a sum of quaternary products of Gram matrix entries, may be computed from the right-hand side, which is a sum involving ternary products of these entries at most.

On the other hand, from (60) and (64), the following result is obtained.

**Proposition 6**  (Doppler-weighted sum of the squared inner tetrahedrons volumes and squared triangle areas)**.** *The following relation holds:*

(70) $$\sum_{A<B<C}^{k}{\left(f_{A}f_{B}f_{C}\right)}^{2}\left[{\left(6V_{UABC}\right)}^{2}+{\left(2A_{ABC}\right)}^{2}\right]=\frac{\lambda }{3}+2\Sigma -K\;\sigma$$

*with $\lambda \equiv \mathrm{tr}G^{3}$, $\sigma \equiv \mathrm{tr}G^{2}$.*

In the next section we show that (70) and (69) are, respectively, the numerator and the denominator of the squared *FGDOP* scalar.

## 6. The FGDOP Matrix $G^{-1}$ and the *FGDOP* Scalar, $\sqrt{\mathrm{tr}G^{-1}}$

The FGDOP matrix $G^{-1}$ is defined as the inverse of the Doppler geometric matrix *G* given by (28). Notice that $G^{-1}$ is a lineal combination of powers of *G*, whose coefficients are polynomial scalars which depend on the traces of the powers of *G* up to fourth order. This is a consequence of the Cayley–Hamilton theorem [53], which assures that *G* satisfies its own characteristic equation

(71) $$G^{4}-\alpha G^{3}+\frac{1}{2}\left(\alpha^{2}-\beta \right)G^{2}-\frac{1}{6}\left(\alpha^{3}-3\alpha \beta +2\gamma \right)G+\left(\det G\right)I=0,$$

with *I* the 4 × 4 unit matrix. The determinant of *G* is given by

(72) $$4!\det G=\alpha^{2}\left(\alpha^{2}-6\beta \right)+8\alpha \gamma +3\beta^{2}-6\delta ,$$

where the notation

(73) $$\alpha \equiv \mathrm{tr}G,\;\beta \equiv \mathrm{tr}G^{2},\;\gamma \equiv \mathrm{tr}G^{3},\;\delta \equiv \mathrm{tr}G^{4}$$

is used. Assuming that *G* is regular, we have the following expression for $G^{-1}$:

(74) $$G^{-1}=-\frac{1}{\det G}\left\{G^{3}-\alpha \;G^{2}+\frac{1}{2}\left(\alpha^{2}-\beta \right)G-\frac{1}{6}\left(\alpha^{3}-3\alpha \beta +2\gamma \right)I\right\}.$$

The *FGDOP* scalar is defined as $\sqrt{\mathrm{tr}G^{-1}}$. From (74) we have

(75) $$\mathrm{tr}G^{-1}=\frac{1}{6\det G}\left(\alpha^{3}-3\alpha \beta +2\gamma \right).$$

In order to obtain a covariant expression for the *FGDOP* scalar, we just need to calculate the trace of the four first powers of *G* (all of them involved in the calculation of detG). The calculations are performed through the Gram matrix G and are presented in Appendix C. The expressions for the traces of *G* are summarized as follows.

**Proposition 7**  (Traces of the powers of *G*)**.** *For k emitters, the traces of the power of the geometric tensor G (38) are given by*

(76) $$\begin{array}{ccc} \alpha \equiv \mathrm{tr}G\;\; & = & 2K\;,\; \end{array}$$

(77) $$\begin{array}{ccc} \beta \equiv \mathrm{tr}G^{2} & = & \sigma +4S+4K^{2}\;,\; \end{array}$$

(78) $$\begin{array}{ccc} \gamma \equiv \mathrm{tr}G^{3} & = & \lambda +6\Sigma +12KS+8K^{3}\;\; \end{array}$$

(79) $$\begin{array}{ccc} \;\delta \equiv \mathrm{tr}G^{4} & = & \omega +8\Lambda +8S^{2}+16K\Sigma +32K^{2}S+16K^{4}. \end{array}$$

*where quantities σ, λ, ω, S, *Σ*, and *Λ* are computed from the angular separation between emitters and the frequency ratios through Equations (52)–(58).*

### 6.1. Determinant of G

Substituting in (72) the values of *α*, *β*, *γ*, and *δ* given by Proposition 7, we obtain, after some elemental cancellations, the following expression for the determinant of the geometric matrix:

(80) $$\det G=\frac{1}{4}\left(\frac{\sigma^{2}}{2}-\omega \right)+2\left[K\frac{\lambda }{3}+S\frac{\sigma }{2}-\Lambda \right].$$

Notice that (80) contains the fourth-order term $\omega =\mathrm{tr}G^{4}$. Nevertheless, as remarked after Proposition 5, this term can be expressed as a combination of lower-order terms according to identity (69). Thus, detG is more easily computed according to the following result.

**Proposition 8**  (Determinant of *G*)**.** *The determinant of the geometric matrix is given by*

(81) $$\det G=K\frac{\lambda }{3}+S\frac{\sigma }{2}-\Lambda =\sum_{A<B<C<D}^{k}{\left(6f_{A}f_{B}f_{C}f_{D}V_{ABCD}\right)}^{2},$$

*which involves the powers of the Gram matrix G until third order.*

### 6.2. The FGDOP Scalar, $\sqrt{\mathrm{tr}G^{-1}}$

To obtain the *FGDOP* scalar, let us take the trace of Equation (74) yielding

(82) $$\mathrm{tr}G^{-1}=\frac{1}{6\det G}\left(\alpha^{3}-3\alpha \beta +2\gamma \right)=\frac{1}{\det G}\left(\frac{\lambda }{3}+2\Sigma -K\sigma \right),$$

where the second equality follows from substituting α=2K and the values of *β* and *γ* given in Appendix C by (A23) and (A25) and later simplifying, and with Σ given by (57). Substituting (80) in (82) we obtain

(83) $$\mathrm{tr}G^{-1}=\frac{\frac{\lambda }{3}+2\Sigma -K\sigma }{K\frac{\lambda }{3}+S\frac{\sigma }{2}-\Lambda }\;,$$

whose square root is the *FGDOP* scalar. In addition, taking into account (69) and (70), we have the following result.

**Proposition 9**  (The FGDOP covariant formula)**.** *The FGDOP scalar at the user location can be evaluated from the following formula:*

(84) $$\begin{array}{c} FGDOP=\sqrt{\frac{\frac{\lambda }{3}+2\Sigma -K\sigma }{K\frac{\lambda }{3}+S\frac{\sigma }{2}-\Lambda }}\;, \end{array}$$

*where $K=\sum_{A=1}^{k}f_{A}^{2}$, $\lambda \equiv \mathrm{tr}G^{3}$, $\sigma \equiv \mathrm{tr}G^{2}$, $S\equiv \sum_{A,B=1}^{k}f_{A}f_{B}G_{AB}$, $\Sigma \equiv \sum_{A,B=1}^{k}f_{A}f_{B}{\left(G^{2}\right)}_{AB}$ and $\Lambda =\sum_{A,B=1}^{k}f_{A}f_{B}{\left(G^{3}\right)}_{AB}$ are computed from the elements of the Gram matrix G, which are given by the frequency ratios $f_{A}$ and the angle $\theta_{AB}$ between emitters, $G_{AB}=f_{A}f_{B}\left(-1+\cos\theta_{AB}\right)$, with A,B=1,…,k and k the number of satellites in view.*

*According to (70) and (81), Equation (84) can be expressed in terms of the areas $A_{ABC}$ and volumes $V_{UABC}$ and $V_{ABCD}$ (see Proposition 3):*

(85) $$\begin{array}{c} FGDOP=\sqrt{\frac{\sum_{A<B<C}^{k}{\left(f_{A}f_{B}f_{C}\right)}^{2}\left[{\left(6V_{UABC}\right)}^{2}+{\left(2A_{ABC}\right)}^{2}\right]}{\sum_{A<B<C<D}^{k}{\left(6f_{A}f_{B}f_{C}f_{D}V_{ABCD}\right)}^{2}}}. \end{array}$$

Massat and Rudnick [15] seem to be the first authors who obtained a geometric formula for the GDOP scalar in the case of four emitters by expressing it in terms of volumes and areas defined by the relative position of the emitters on the user’s unit celestial sphere (see Figure 2). More recently [16], Santerre, Geiger, and Banville have generalized Massat and Rudnick’s results for an arbitrary number of emitters. The geometric interpretation of the GDOP scalar is here recovered from (84) and (85), considering all frequency ratios equal to one, $f_{A}=1_{A}$, which corresponds to the static situation where emitters and user are relatively at rest.

**Proposition 10**  (Geometric interpretation of the FGDOP for unit frequency ratios)**.** *In terms of the volumes and areas associated to the relative positions of the quads and triads of emitters on the unit celestial sphere $S_{U}$ of the user U (see Figure 2), for $f_{A}=1$ (for any emitter), the FGDOP formulae (84) and (85) admit the following expression and geometric interpretation for a configuration of k emitters in view:*

(86) GDOP=λ3+2Σ−kσkλ3+Sσ2−Λ=∑i=1k3[(Ui)2+19(Ai)2]∑i=1k4(Vi)2.

*where*

- *$V_{i}$ is the volume of the inscribed tetrahedron defined by quad i of emitters.*
- *$U_{i}$ is the volume of the inner tetrahedron defined by triad i of emitters and the user.*
- *$A_{i}$ is the area of the triangle defined by triad i of emitters.*

## 7. Applying the Covariant GDOP Formula

We have shown that the Doppler GDOP scalar can be directly computed from (84), which becomes (86) when $f_{A}=1$ for all emitters. A practical advantage of this formula is that it only involves the calculation of the Gram matrix G of *k* null vectors and its second and third powers. Indeed, the calculations simplify remarkably when we consider the symmetric configurations of satellites in view. The essential symmetric emitter configurations are the Platonic ones, which correspond to k=4,6,8,12, or 20 emitters placed on the vertices of a regular tetrahedron, octahedron, cube, icosahedron, or dodecahedron, respectively, inscribed on $S_{U}$. For *k* emitters, it is known that the lowest GDOP value is $\sqrt{10/k}$ and that this value is attained by Platonic (and other symmetric) configurations [50]. As shown in [49], the Gram matrix of a Platonic configuration with *k* emitters satisfies the minimal equation $G^{3}+\frac{2}{3}kG^{2}-\frac{k^{2}}{3}G=0$. In fact, for each Platonic situation, G is a symmetric positive definite k×k matrix with the following eigenvalues: −k (simple eigenvalue), k/3 (triple eigenvalue), and 0 (eigenvalue of multiplicity k−4). Or, equivalently, for any Platonic configuration, the vector axis vanishes, e=0, and the tensor axis is isotropic, $E=\frac{k}{3}I$, with *I* the 3×3 unit matrix. In this section, we present exact GDOP calculations for symmetric configurations of a reduced number of emitters (four or five).

### 7.1. Symmetric Configurations with Four Emitters

As a clarifying example, let us consider the most simple situation: four static emitters forming a symmetric configuration around a static user located at the origin of an inertial coordinate system.

(i) The emitters are placed at the vertices of a regular tetrahedron: (1,1,1), (1,−1,−1), (−1,1,−1), (−1,−1,1). The signals propagate along the null directions defined by the four 4-vectors:
  (87) $$\begin{array}{ccc} l_{1} & = & (1,\frac{1}{\sqrt{3}},\frac{1}{\sqrt{3}},\frac{1}{\sqrt{3}})\;,\;\; \end{array}$$
  (88) $$\begin{array}{ccc} l_{2} & = & (1,\frac{1}{\sqrt{3}},-\frac{1}{\sqrt{3}},-\frac{1}{\sqrt{3}})\;, \end{array}$$
  (89) $$\begin{array}{ccc} l_{3} & = & (1,-\frac{1}{\sqrt{3}},\frac{1}{\sqrt{3}},-\frac{1}{\sqrt{3}})\;, \end{array}$$
  (90) $$\begin{array}{ccc} l_{4} & = & (1,-\frac{1}{\sqrt{3}},-\frac{1}{\sqrt{3}},\frac{1}{\sqrt{3}})\;, \end{array}$$
  expressed in an orthonormal basis $\left\{e_{0},e_{1},e_{2},e_{3}\right\}$ adapted to the user, that is, with $e_{0}=u$. In this case, the geometric matrix is diagonal, $G=diag(4,\frac{4}{3},\frac{4}{3},\frac{4}{3})$, with $\det G=\frac{4^{4}}{3^{3}}$ ($V=\frac{8}{9\sqrt{3}}$), and the GDOP scalar is $\sqrt{\mathrm{tr}G^{-1}}=\sqrt{2.5}$. All the observation angles are equal, $n_{A}\cdot n_{B}=\cos\theta_{AB}\equiv c_{AB}=-\frac{1}{3}$ for all A≠B, and the angular identity (A6) is satisfied (see Appendix A). The basis $\left\{l_{1},l_{2},l_{3},l_{4}\right\}$ is a null symmetric frame [35,54,55], $l_{A}\cdot l_{B}=-\frac{4}{3}$ for all A≠B, and the Gram matrix is
  (91) $${\left(G\right)}_{AB}=-\frac{4}{3}\left(\begin{array}{cccc} 0 & 1 & 1 & 1\\ 1 & 0 & 1 & 1\\ 1 & 1 & 0 & 1\\ 1 & 1 & 1 & 0 \end{array}\right).$$
(ii) The emitters are placed at the vertices of an iso-rectangular tetrahedron: (0,0,1), (1,0,0), $\left(-\frac{1}{2},\frac{\sqrt{3}}{2},0\right)$, $\left(-\frac{1}{2},-\frac{\sqrt{3}}{2},0\right)$. The null vectors
  (92) $$\begin{array}{ccc} l_{1} & = & (1,0,0,1)\;,\;\;\; \end{array}$$
  (93) $$\begin{array}{ccc} l_{2} & = & (1,1,0,0)\;,\;\;\; \end{array}$$
  (94) $$\begin{array}{ccc} l_{3} & = & (1,-\frac{1}{2},\frac{\sqrt{3}}{2},0)\;,\;\; \end{array}$$
  (95) $$\begin{array}{ccc} l_{4} & = & (1,-\frac{1}{2},-\frac{\sqrt{3}}{2},0)\;, \end{array}$$
  allow us to construct the geometric matrix and the Gram matrix,
  $${\left(G\right)}_{AB}=\left(\begin{array}{cccc} 4 & 0 & 0 & 1\\ 0 & \frac{3}{2} & 0 & 0\\ 0 & 0 & \frac{3}{2} & 0\\ 1 & 0 & 0 & 1 \end{array}\right),\;{\left(G\right)}_{AB}=-\left(\begin{array}{cccc} 0 & 1 & 1 & 1\\ \;1 & 0 & \frac{3}{2} & \frac{3}{2}\\ \;1 & \frac{3}{2} & 0 & \frac{3}{2}\\ \;1 & \frac{3}{2} & \frac{3}{2} & 0 \end{array}\right),$$
  which are regular with $\det G=\frac{27}{4}=-\det G$ ($V=\frac{\sqrt{3}}{4}$). In this configuration the GDOP scalar is $\sqrt{\mathrm{tr}G^{-1}}=\sqrt{3}$. The observation angles $c_{AB}$ are $c_{12}=c_{13}=c_{14}=0$, and $c_{23}=c_{24}=c_{34}=-\frac{1}{2}$, which satisfy the constraint (A6).
  Table 1 summarizes calculations for symmetric configurations of four emitters.

### 7.2. Symmetric Configurations with Five Emitters

Let us consider two symmetric configurations with five static emitters: one with *e* = 0 and E∝I and the other with e≠0 and E≁I (see (33)). Notice that a pentahedron is not a Platonic configuration, that is, there is no five-satellite configuration that has both the vector axis equal to zero and an isotropic tensor axis.

(i) The emitters are placed at the following vertices of a pentahedron: (0,0,1), (1,0,0), $\left(-1/2,\sqrt{3}/2,0\right)$, $\left(-1/2,-\sqrt{3}/2,0\right)$, (0,0,−1). In this case, the geometric matrix is diagonal, $G=diag(5,\frac{3}{2},\frac{3}{2},2)$. The vector axis is zero and the tensor axis has one simple and one double eigenvalue. The GDOP scalar is $\sqrt{\mathrm{tr}G^{-1}}=\sqrt{61/30}\approx 1.426$.
(ii) The emitters are placed at these vertices of a pentahedron: (0,0,1), $\frac{1}{\sqrt{6}}\left(\sqrt{5},0,-1\right)$, $-\frac{1}{\sqrt{6}}\left(\sqrt{5},0,1\right)$, $\frac{1}{\sqrt{6}}\left(0,\sqrt{5},-1\right)$, $-\frac{1}{\sqrt{6}}\left(0,\sqrt{5},1\right)$. In this case, which is also considered in [50], the geometric matrix is non-diagonal,
  $${\left(G\right)}_{AB}=\left(\begin{array}{cccc} 5 & 0 & 0 & 1-\frac{4}{\sqrt{6}}\\ 0 & \frac{5}{3} & 0 & 0\\ 0 & 0 & \frac{5}{3} & 0\\ 1-\frac{4}{\sqrt{6}} & 0 & 0 & \frac{5}{3} \end{array}\right).$$
  The vector axis is $e=\left(1-\frac{4}{\sqrt{6}}\right)\left(0,0,1\right)\neq 0$, and the tensor axis has a triple eigenvalue equal to 5/3. The GDOP scalar is $\sqrt{\mathrm{tr}G^{-1}}\approx 1.428$, which is slightly above the GDOP value of situation (i).

### 7.3. RPS with One Inertial and Three Static Emitters

Recently, RPSs with different configurations of four emitters in Minkowski space-time have been analysed in detail [42]. The simplest non-static RPS configuration considered in that reference involves one emitter moving with constant velocity and three static emitters. We will use this example to illustrate how the FGDOP concept is incorporated into GDOP computations.

In this simple configuration emitters 2, 3 and 4 are static with respect to an inertial observer $e_{0}$ ($e_{0}^{2}=-1$). Emitter 1 is moving with constant velocity $|\beta_{1}|\;=\frac{1}{2}$ with respect to $e_{0}$ along the spatial main bisectrix—note that, since we are using units in which the speed of light in vacuum is *c* = 1, emitter 1 is moving with a highly relativistic velocity with respect to the inertial observer $e_{0}$—in such a way that, at proper time $\tau^{1}=0$, the four emitters spatially form a regular tetrahedron of vertices (1,1,1), (1,−1,−1), (−1,1,−1), and (−1,−1,1).

The emitters’ world-lines $\gamma_{A}$ (A=1,2,3,4) are written as follows—for clarity, for any space-time vector living in the space orthogonal to $e_{0}$, i.e., $E_{⊥}$, we will abuse the notation and use a three-dimensional vector (as in (1,1,1)) when, in fact, it is a four-dimensional space-time vector with a vanishing first coordinate (such as (0,1,1,1))— taking into account that the relativistic factor is $w_{1}={\left(1-\beta_{1}^{2}\right)}^{-\frac{1}{2}}=\frac{2}{\sqrt{3}}$ (see [42]):

(96) $$\begin{array}{cccc} \gamma_{1}\left(\tau^{1}\right)= & \frac{2}{\sqrt{3}}\tau^{1}\left[e_{0}+\frac{1}{2\sqrt{3}}\left(1,1,1\right)\right]+\left(1,1,1\right),\; & \gamma_{2}\left(\tau^{2}\right) & =\tau^{2}\;e_{0}+\left(1,-1,-1\right),\\ \gamma_{3}\left(\tau^{3}\right)= & \tau^{3}\;e_{0}+\left(-1,1,-1\right),\; & \gamma_{4}\left(\tau^{4}\right) & =\tau^{4}\;e_{0}+\left(-1,-1,1\right). \end{array}$$

We can calculate the FGDOP scalar for a user trying to locate itself using this RPS. The user is static with respect to the inertial observer $e_{0}$ and receives the emission coordinates $\tau^{1}=-\frac{1}{2}$, $\tau^{2}=\tau^{3}=\tau^{4}=\frac{1}{2}$. According to Table 1 of [42], the user’s inertial coordinates are as follows:

(97) $$x^{0}=\frac{9}{44}\left(5\sqrt{3}+3\right),\;x^{1}=x^{2}=x^{3}=-\frac{1}{44}\left(9\sqrt{3}+23\right).$$

Since emitters 2, 3, and 4 are static, the corresponding frequency ratios are $f_{2}=f_{3}=f_{4}=1$. To calculate $f_{1}$ using the first equality in (10), since the user’s four-velocity is $u=e_{0}$, we need to calculate the four-velocity of emitter 1 at the emission event, $u_{1}\left(-\frac{1}{2}\right)$, and the light-like four-vector $m_{1}=x-\gamma_{1}$, where *x* is the user’s position four-vector at the reception event given by (97) and $\gamma_{1}$ the position four-vector of emitter 1 at the emission event, $\gamma_{1}\left(-\frac{1}{2}\right)$.

Differentiating $\gamma_{1}$ (96) with respect to $\tau^{1}$ we obtain $u_{1}$:

(98) $$u_{1}=\frac{2}{\sqrt{3}}e_{0}+\frac{1}{3}\left(1,1,1\right).$$

Calculating the difference $x-\gamma_{1}$ we obtain $m_{1}$ and $n_{1}$ (8):

(99) $$m_{1}=\frac{\left(81+179\sqrt{3}\right)}{132}\left(e_{0}+n_{1}\right),\;n_{1}=-\frac{1}{\sqrt{3}}\left(1,1,1\right).$$

Now we compute the scalar products $m_{1}\cdot u$ and $m_{1}\cdot u_{1}$ to obtain $f_{1}$ (10):

(100) $$\begin{array}{c} m_{1}\cdot u=-\frac{1}{132}\left(81+179\sqrt{3}\right),\;m_{1}\cdot u_{1}=-\frac{\sqrt{3}}{132}\left(81+179\sqrt{3}\right), \end{array}$$

(101) $$\begin{array}{c} f_{1}=\frac{1}{\sqrt{3}}. \end{array}$$

Analogously, we compute $m_{a}=x-\gamma_{a}$ and $n_{a}$ (a=2,3,4):

(102) $$\begin{array}{cc} m_{a} & =\frac{5(1+9\sqrt{3})}{44}\left(e_{0}+n_{a}\right),\;a=2,3,4,\;\;\;\; \end{array}$$

(103) $$\begin{array}{cc} n_{2} & =-\frac{1}{5(1+9\sqrt{3})}\left(67+9\sqrt{3},3\left(-7+3\sqrt{3}\right),3\left(-7+3\sqrt{3}\right)\right),\; \end{array}$$

(104) $$\begin{array}{cc} n_{3} & =-\frac{1}{5(1+9\sqrt{3})}\left(3\left(-7+3\sqrt{3}\right),67+9\sqrt{3},3\left(-7+3\sqrt{3}\right)\right),\; \end{array}$$

(105) $$\begin{array}{cc} n_{4} & =-\frac{1}{5(1+9\sqrt{3})}\left(3\left(-7+3\sqrt{3}\right),3\left(-7+3\sqrt{3}\right),67+9\sqrt{3}\right). \end{array}$$

We are ready to compute the Frequency Geometric Matrix (28):

(106) $$\begin{array}{c} {\left(G\right)}_{AB}=\left(\begin{array}{cccc} \frac{10}{3} & q & q & q\\ q & \frac{10}{9} & r & r\\ q & r & \frac{10}{9} & r\\ q & r & r & \frac{10}{9} \end{array}\right),\\ q=-\frac{8}{495}\left(36+17\sqrt{3}\right),\;r=\frac{2}{27225}\left(-2443+1296\sqrt{3}\right), \end{array}$$

and the Gram matrix with elements $G_{AB}=l_{A}\cdot l_{B}$, where $l_{A}=f_{A}\left(e_{0}+n_{A}\right)$:

(107) $$\begin{array}{c} {\left(G\right)}_{AB}=\left(\begin{array}{cccc} 0 & s & s & s\\ s & 0 & t & t\\ s & t & 0 & t\\ s & t & t & 0 \end{array}\right),\;s=\frac{1}{165}\left(32-46\sqrt{3}\right),\;t=\frac{32}{3025}\left(-122+9\sqrt{3}\right). \end{array}$$

To calculate the FGDOP scalar, we can directly use $\sqrt{\mathrm{tr}G^{-1}}$ and (106) or we can apply Proposition 9 using (107). In both cases one obtains FGDOP≈4.405 (numerical computations were performed using Mathematica software version 13.3).

For comparison with the static case, we compute the classical Geometric matrix (25) setting $f_{1}=1$:

(108) $$\begin{array}{c} {\left(G_{c}\right)}_{AB}=\left(\begin{array}{cccc} 4 & q_{c} & q_{c} & q_{c}\\ q_{c} & \frac{4}{3} & r_{c} & r_{c}\\ q_{c} & r_{c} & \frac{4}{3} & r_{c}\\ q_{c} & r_{c} & r_{c} & \frac{4}{3} \end{array}\right),\\ q_{c}=-\frac{2}{165}\left(48+41\sqrt{3}\right),\;r_{c}=\frac{4}{9075}\left(97+216\sqrt{3}\right). \end{array}$$

Calculating the (static) GDOP scalar using $\sqrt{\mathrm{tr}G_{c}^{-1}}$, one obtains GDOP≈3.070. This value is significantly less than the FGDOP scalar, as expected considering that emitter 1 is moving away from the user.

### 7.4. RPS with Four Rotating Emitters

Following the same approach of the previous section, we will now compute the FGDOP scalar for a configuration of four rotating emitters. A similar configuration was analysed in [42]. In this case, however, the fourth emitter, instead of being static, will also rotate.

In this configuration emitters 1, 2 and 3 follow uniform circular motion on the spatial {x,y} plane at a constant radial distance $r_{0}$ ($r_{0}>0$), with constant angular velocity Ω with respect to the inertial observer $e_{0}$, equally spaced at an angle of $\frac{2\pi }{3}$. Emitter 4 also follows uniform circular motion with constant angular velocity Ω, but on the spatial {y,z} plane at a constant radial distance *d* (d>0). The emitters’ world-lines $\gamma_{A}$ (A=1,2,3,4) are then written as follows:

(109) $$\begin{array}{cc} \gamma_{1}= & w\;\tau^{1}\;e_{0}+r_{0}\left(\cos\left(\Omega w\tau^{1}\right),\sin\left(\Omega w\tau^{1}\right),0\right),\\ \gamma_{2}= & w\;\tau^{2}\;e_{0}+r_{0}\left(\cos\left(\Omega w\tau^{2}-\frac{2\pi }{3}\right),\sin\left(\Omega w\tau^{2}-\frac{2\pi }{3}\right),0\right),\\ \gamma_{3}= & w\;\tau^{3}\;e_{0}+r_{0}\left(\cos\left(\Omega w\tau^{3}-\frac{4\pi }{3}\right),\sin\left(\Omega w\tau^{3}-\frac{4\pi }{3}\right),0\right),\\ \gamma_{4}= & w_{4}\;\tau^{4}\;e_{0}+d\left(0,\sin\left(\Omega w_{4}\tau^{4}\right),\cos\left(\Omega w_{4}\tau^{4}\right)\right), \end{array}$$

where $w\equiv w_{1}=w_{2}=w_{3}={\left(1-\beta^{2}\right)}^{-\frac{1}{2}}$, with $\beta =\Omega \;r_{0}$, and $w_{4}={\left(1-\beta_{4}^{2}\right)}^{-\frac{1}{2}}$, with $\beta_{4}=\Omega \;d$, are the relativistic factors.

We can calculate the FGDOP scalar for a user trying to locate themself using this RPS. For simplification, the user is static with respect to the inertial observer $e_{0}$ and receives the emission coordinates

(110) $$\tau^{1}=\tau^{2}=\tau^{3}=-\frac{r_{0}}{w},\;\tau^{4}=-\frac{d}{w_{4}}.$$

These emission coordinates correspond to the following user’s inertial coordinates:

(111) $$x^{0}=x^{1}=x^{2}=x^{3}=0.$$

We will now calculate the frequency ratios $f_{A}$ using the first equality in (10). Since the user’s four-velocity is $u=e_{0}$, we need to calculate the emitters’ four-velocities at the emission events, $u_{1}\left(-\frac{r_{0}}{w}\right)$, $u_{2}\left(-\frac{r_{0}}{w}\right)$, $u_{3}\left(-\frac{r_{0}}{w}\right)$ $u_{4}\left(-\frac{d}{w_{4}}\right)$, and the light-like vectors $m_{A}$. As the user is located at the temporal and spatial origin (111), the light-like vectors are simply $m_{A}=-\gamma_{A}$, with $\gamma_{A}$ the position four-vector of emitter *A* at the respective emission event (110).

To obtain $u_{A}$ at the emission event, we evaluate the derivative of $\gamma_{A}$ (109) with respect to $\tau^{A}$ at the respective emission event (110):

(112) $$\begin{array}{cc} u_{1} & =w\;e_{0}+w\;\Omega r_{0}\left(\sin\left(\Omega r_{0}\right),\cos\left(\Omega r_{0}\right),0\right),\\ u_{2} & =w\;e_{0}+w\;\Omega r_{0}\left(\sin\left(\Omega r_{0}+\frac{2\pi }{3}\right),\cos\left(\Omega r_{0}+\frac{2\pi }{3}\right),0\right),\\ u_{3} & =w\;e_{0}+w\;\Omega r_{0}\left(\sin\left(\Omega r_{0}+\frac{4\pi }{3}\right),\cos\left(\Omega r_{0}+\frac{4\pi }{3}\right),0\right),\\ u_{4} & =w_{4}\;e_{0}+w_{4}\;\Omega d\left(0,\cos(\Omega d),\sin(\Omega d)\right). \end{array}$$

Now we compute $m_{A}$ and $n_{A}$ (8)

(113) $$\begin{array}{cccc} m_{1} & =r_{0}\left(e_{0}+n_{1}\right), & \;n_{1} & =\left(-\cos\left(\Omega r_{0}\right),\sin\left(\Omega r_{0}\right),0\right)\\ m_{2} & =r_{0}\left(e_{0}+n_{2}\right), & \;n_{2} & =\left(-\cos\left(\Omega r_{0}+\frac{2\pi }{3}\right),\sin\left(\Omega r_{0}+\frac{2\pi }{3}\right),0\right),\\ m_{3} & =r_{0}\left(e_{0}+n_{3}\right), & \;n_{3} & =\left(-\cos\left(\Omega r_{0}+\frac{4\pi }{3}\right),\sin\left(\Omega r_{0}+\frac{4\pi }{3}\right),0\right),\\ m_{4} & =d\left(e_{0}+n_{4}\right), & \;n_{4} & =\left(0,\sin(\Omega d),-\cos(\Omega d)\right), \end{array}$$

and calculate the scalar products $m_{A}\cdot u$ and $m_{A}\cdot u_{A}$ to obtain $f_{A}$ (10):

(114) $$\begin{array}{cccccc} m_{a}\cdot u & =-r_{0}, & \;m_{a}\cdot u_{a} & =-w\;r_{0}, & \;f & \equiv f_{a}=\frac{1}{w},\;\left(a=1,2,3\right),\\ m_{4}\cdot u & =-d, & \;m_{4}\cdot u_{4} & =-w_{4}\;d, & \;f_{4} & =\frac{1}{w_{4}}. \end{array}$$

We are ready to compute the Frequency Geometric Matrix (28)

(115) $${\left(G\right)}_{AB}=\left(\begin{array}{cccc} 3f^{2}+f_{4}^{2} & 0 & f_{4}^{2}\sin\left(\Omega d\right) & -f_{4}^{2}\cos\left(\Omega d\right)\\ 0 & \frac{3}{2}f^{2} & 0 & 0\\ f_{4}^{2}\sin\left(\Omega d\right) & 0 & \frac{3}{2}f^{2}+f_{4}^{2}\sin^{2}\left(\Omega d\right) & -\frac{1}{2}f_{4}^{2}\sin\left(2\Omega d\right)\\ -f_{4}^{2}\cos\left(\Omega d\right) & 0 & -\frac{1}{2}f_{4}^{2}\sin\left(2\Omega d\right) & f_{4}^{2}\cos^{2}\left(\Omega d\right) \end{array}\right),$$

and the Gram matrix with elements $G_{AB}=l_{A}\cdot l_{B}$, where $l_{A}=f_{A}\left(e_{0}+n_{A}\right)$:

(116) $${\left(G\right)}_{AB}=\left(\begin{array}{cccc} 0 & p & p & q\\ p & 0 & p & r\\ p & p & 0 & s\\ q & r & s & 0 \end{array}\right),$$

(117) $$\begin{array}{ll} p=-\frac{3}{2}f^{2}, & q=ff_{4}\left(-1+\sin\left(\Omega r_{0}\right)\sin\left(\Omega d\right)\right),\\ r=ff_{4}\left(-1+\sin\left(\Omega r_{0}+\frac{2\pi }{3}\right)\sin\left(\Omega d\right)\right), & s=ff_{4}\left(-1+\sin\left(\Omega r_{0}+\frac{4\pi }{3}\right)\sin\left(\Omega d\right)\right). \end{array}$$

To calculate the FGDOP scalar, we can use $\sqrt{\mathrm{tr}G^{-1}}$ and (115) or we can apply Proposition 9 using (116). In both cases, one obtains

(118) $$FGDOP=w\sqrt{2+\frac{w_{4}^{2}}{w^{2}}+\left(1+\frac{w_{4}^{2}}{w^{2}}\right)\tan^{2}\left(\Omega d\right)}.$$

This example shows how the transverse relativistic Doppler effect is taken into account in the FGDOP metric. In fact, for a constellation of emitters in circular orbits around a common centre and a static receiver located at that centre, the Doppler effect is purely relativistic and transverse. In particular, when $w=w_{4}$, Equation (118) becomes

(119) $$FGDOP=w\sqrt{3+2\tan^{2}\left(\Omega d\right)}.$$

Therefore, the transverse Doppler effect increases the classical GDOP.

For comparison, the classical Geometric matrix (25) can be directly obtained by setting $f=f_{4}=1$ in (115):

(120) $${\left(G\right)}_{AB}=\left(\begin{array}{cccc} 4 & 0 & \sin(\Omega d) & -\cos(\Omega d)\\ 0 & \frac{3}{2} & 0 & 0\\ \sin(\Omega d) & 0 & \frac{3}{2}+\sin^{2}\left(\Omega d\right) & -\frac{1}{2}\sin\left(2\Omega d\right)\\ -\cos(\Omega d) & 0 & -\frac{1}{2}\sin\left(2\Omega d\right) & \cos^{2}\left(\Omega d\right) \end{array}\right),$$

yielding the following expression for the GDOP scalar:

(121) $$GDOP=\sqrt{3+2\tan^{2}\left(\Omega d\right)},$$

which is also recovered from (118) setting $w=w_{4}=1$. If we particularize this expression for Ω = 0, we recover the GDOP scalar value of $\sqrt{3}$ obtained in Section 7.1 for the static iso-rectangular tetrahedron. Furthermore, whenever Ωd is an odd multiple of $\frac{\pi }{2}$, the four emitters are coplanar on the {x,y} plane, hence the GDOP scalar tends to infinity.

### 7.5. RPS Simulations: An Invitation

With a view to more realistic scenarios, the previous examples illustrate how the FGDOP concept is numerically implemented. In the current GNSS and LEO simulations, Walker satellite configurations [56] are used (as basic elements). For instance, in [50], global GDOP maps on the Earth surface are discussed by comparing different Walker configurations, and in [21], the authors consider a LEO constellation simulated by a Walker configuration and supplementary near-polar satellite orbits, analysing the Cramér–Rao lower bound for Doppler-assisted positioning errors. On the other hand, References [43,44,45,46] present GNSS simulations and error positioning analyses (in the vicinity of the Earth) based on nominal GALILEO satellite trajectories, applying the transformation from emission to inertial coordinates of relativistic positioning. Simulations including the FGDOP concept require robust research of basic emitter configurations in RPS theory [38,39].

The symmetric examples presented in Section 7.2 aim to show how Equation (86) allows us to calculate the scalar GDOP without matrix inversion. This algebraic formula also applies to any given configuration of emitters, regardless of the shape or symmetry (if any) of the configuration. Static symmetric configurations are of particular interest for positioning. In fact, they are used in real-world positioning situations where the satellites are replaced by beacons. Pulsars are excellent examples of astrophysical beacons. Recently, a Monte Carlo Optimization Algorithm [57] has been proposed to optimize the GDOP in an Incremental GDOP model considering hybrid (static and movable) systems of pseudolites. The algorithm applies the geometric interpretation of the GDOP established in [16] and revisited in Proposition 10. The application of Equation (86) to GNSS and LEO constellations is still pending. However, this task requires a deeper understanding of numerical techniques used in previous numerical RPS computations:

1. To determine the coordinate transformation from emission to inertial coordinates. This provides the space-time user localization and the geometry of the RPS regions and coordinate domains.
2. To obtain, for each event of a RPS coordinate domain, the relative elements appearing in the FGDOP formula (frequency ratios and angular separations).
3. To compute Equation (86) at each event from the previously obtained data.
4. To generate a map of the FGDOP scalar on the coordinate domains of the RPS.

We highly value Professor Diego Pascual Sáez Milan’s contribution to the development of numerical RPS theory. References [43,44,45] initiate a line of work that invites us to take the numerical approach further to achieve a realistic implementation of RPS theory in current GNSSs and other satellite constellations.

Specifically, in Ref. [43] by Puchades and Sáez, a realistic experimental setup is presented: the positioning of GPS (Galileo) satellites using the Galileo (GPS) constellation. The analysis is conducted within the framework of RPS theory. Satellite trajectories are modelled as circular orbits in Schwarzschild geometry, and the propagation of the broadcast signals is modelled using the geometric optics approximation: to leading order, transmitted electromagnetic signals follow straight lines in flat space-time. Thus, the satellite orbits belong to different Walker configurations, and their world-lines (referred to a quasi-inertial observer $e_{0}$) are described as

$$\gamma_{s}\left(\tau^{s}\right)=\Gamma_{s}\tau^{s}e_{0}+R_{s}\;\vec{s},$$

where the index *s* refers to the satellite; $\tau^{s}$, $\Gamma_{s}$, and $R_{s}$ represent the proper time, the relativistic factor, and the orbital radius, respectively; and $\vec{s}$ denotes the unit vector from the common centre of the circular orbits to the satellite *s*, conveniently parametrized using standard Walker notation. A realistic simulation of this experimental setup, including FGDOP calculations, requires the numerical implementation of steps 1 through 4 (mentioned above) and lies beyond the initial scope of this work.

## 8. Summary and Discussion

For the sake of conciseness, the main results of the paper are presented as propositions: the geometric interpretation of the Jacobian determinant J of the coordinate transformation from inertial to emission coordinates (Proposition 1); the tensor expression of the Frequency Geometric Matrix *G* for *k* emitters (Proposition 2); the geometric interpretation of the principal minors of the Gram matrix G of *k* null vectors (Propositions 3 and 4); the frequency-weighted sums of geometric elements on the user’s unit celestial sphere $S_{U}$ and its connection with the computation of the FGDOP (Propositions 5 and 6); the expressions for the traces of the powers of *G* and detG (Proposition 7 and 8); and the closed FGDOP formula and its geometric interpretations (Propositions 9 and 10).

Equation (84) (see Proposition 9) is an analytic formula that can be used to compute the *FGDOP* scalar and provides a closed and exact expression that can be implemented in *Mathematica* to perform numerical calculations and graphic representations of the *FGDOP* scalar for different user locations. Moreover, even in a situation with static emitters (lighthouses, beacons or pseudolites) where all the frequency ratios are equal to one, the application of our formula improves the GDOP estimation procedures that are employed in the current GNSS based on pseudorange measurements. In GNSSs, a similar situation occurs when the user location and clock offset are obtained from an exact algebraic formula [58,59]. For an observation of very short duration (i.e an epoch in GNSS positioning determination) a closed algebraic formula provides the preliminary space-time user location from the received satellite ephemerides, broadcast at the corresponding emission times. Nevertheless, since pseudorange measurements are conceived as range measurements affected by clock offsets and observation errors, exact solutions are used as initial values for the iterative adjustment and optimization methods [60].

Neither the determination of the eigenvalues of *G* nor the numerical computation of the inverse matrix of *G* (the FGDOP matrix $G^{-1}$) are essential to compute the *FGDOP* scalar, which is defined from its trace, $FGDOP=\sqrt{\mathrm{tr}G^{-1}}$. In fact, Equation (84) only requires the computation of the Gram matrix G and its powers $G^{2}$ and $G^{3}$ (the fourth power of G is not necessary to compute the *FGDOP* scalar), as a corollary of Proposition 5. Indeed, the common matrix procedure to calculate the inverse of *G* from its adjoint elements may also be employed, specially in simple static symmetric situations. Nevertheless, by programming Equation (84) in GNSS situations with moving satellites, the computation time required to determine the *FGDOP* scalar can be reduced since only the square and the cube of G need to be computed. Moreover, Equation (84) only involves the frequency ratios and the angles between emitters and can therefore be directly implemented from observational data.

Equation (84) applies for a given user, located at a given space-time event. In a RPS operating in flat space-time, the location of a user is provided by the transformation from emission to inertial coordinates, which, in bifurcation situations, requires additional observational data to determine the sign of the Jacobian determinant (or RPS orientation) [38,39]. In this context, it makes sense to ask the following question: at a given space-time event, how are the *FGDOP* scalars measured at that event by different observers related? In other words, how does the *FGDOP* scalar change under a proper Lorentz transformation? The answer to this question requires a fully relativistic approach to the FGDOP concept, which is developed in [49]. A key result merits advancement here. It may be proved that, for every quad of emitter, there always exists an observer that sees three of these emitters symmetrically distributed on its unit celestial sphere $S_{U}$ (that is, forming the same angle *θ* between them). Then, the general Equation (84) allows us to determine the FGDOP for this user and the expression involves, in addition to frequency ratios, only three angles: *θ* and the two angles that determine the position of the fourth emitter on $S_{U}$.

It must be noted that common errors inherent in navigation systems (such as measurement noise, multipath effects, or satellite orbit errors) cannot be ignored. Nevertheless, since these effects are non-relativistic, they would not represent a true obstacle when moving from a Newtonian to a fully relativistic conception of GNSSs (they may be modelled as Newtonian effects and implemented in the theory as in classical GNSSs).

## Figures and Tables

**Table 1:** Computing the GDOP scalar from invariant quantities. Two highly symmetric configurations of four emitters are considered: a regular and an iso-rectangular tetrahedron.

Tetahedron	*S*	*σ*	Σ	*λ*	Λ	*ω*	detG	$\sqrt{\mathrm{tr}G^{-1}}$
Regular	−16	$\frac{64}{3}$	64	−6detG	$-4^{4}$	$7\cdot \frac{4^{5}}{3^{3}}$	$\frac{4^{4}}{3^{3}}$	$\sqrt{2.5}$
Iso-rectangular	−15	$\frac{39}{2}$	57	−7detG	−216	$\frac{1737}{8}$	$\frac{3^{3}}{2^{2}}$	$\sqrt{3}$

## Data Availability

The original contributions presented in this study are included in the article. Further inquiries can be directed to the corresponding author.

## Appendix A. Proof of Proposition 3

The substitution of (42) in (45) leads to the first relation (48) of Proposition 3. To prove the second relation (49), notice that the squared volume of the parallelepiped defined by the triad of vectors $\left\{n_{A},n_{B},n_{C}\right\}$ is equal to its Gramian determinant, and then,

(A1) $$\begin{array}{ccc} {\left(6V_{UABC}\right)}^{2} & = & {\left(n_{A},n_{B},n_{C}\right)}^{2}=\left|\begin{array}{ccc} 1 & n_{A}\cdot n_{B} & n_{A}\cdot n_{C}\\ n_{A}\cdot n_{B} & 1 & n_{B}\cdot n_{C}\\ n_{A}\cdot n_{C} & n_{B}\cdot n_{C} & 1 \end{array}\right|=\left|\begin{array}{ccc} 1 & c_{AB} & c_{AC}\\ c_{AB} & 1 & c_{BC}\\ c_{AC} & c_{BC} & 1 \end{array}\right|\\ & = & 1+2\;c_{AB}\;c_{AC}\;c_{BC}-\left(c_{AB}^{2}+c_{AC}^{2}+c_{BC}^{2}\right)\\ & = & 2{\bar{G}}_{AB}{\bar{G}}_{AC}{\bar{G}}_{BC}+2\left({\bar{G}}_{AB}{\bar{G}}_{AC}+{\bar{G}}_{AB}{\bar{G}}_{BC}+{\bar{G}}_{AB}{\bar{G}}_{BC}\right)\\ & & -[{\left({\bar{G}}_{AB}\right)}^{2}+{\left({\bar{G}}_{AC}\right)}^{2}+{\left({\bar{G}}_{BC}\right)}^{2}], \end{array}$$

where ${\bar{G}}_{AB}$ is the reduced Gram matrix of $\left\{n_{A},n_{B},n_{C}\right\}$,

(A2) $${\bar{G}}_{AB}=-1+c_{AB},\;c_{AB}=n_{A}\cdot n_{B}=\cos\theta_{AB},\;G_{AB}=f_{A}f_{B}{\bar{G}}_{AB}.$$

On $S_{U}$ the triad $\left\{n_{A},n_{B},n_{C}\right\}$ defines a triangle of side lengths $d_{AB}$, $d_{AC}$, and $d_{BC}$, whose area $A_{ABC}$ satisfies Heron’s formula:

(A3) $$\begin{array}{ccc} {\left(4A_{ABC}\right)}^{2} & \equiv & \left(d_{AB}+d_{AC}+d_{BC}\right)\left(-d_{AB}+d_{AC}+d_{BC}\right)\left(d_{AB}-d_{AC}+d_{BC}\right)\left(d_{AB}+d_{AC}-d_{BC}\right)\\ & = & 2\left(d_{AB}^{2}d_{AC}^{2}+d_{AB}^{2}d_{BC}^{2}+d_{AC}^{2}d_{BC}^{2}\right)-\left(d_{AB}^{4}+d_{AC}^{4}+d_{BC}^{4}\right)\\ & = & 4\left\{2\left({\bar{G}}_{AB}{\bar{G}}_{AC}+{\bar{G}}_{AB}{\bar{G}}_{BC}+{\bar{G}}_{AC}{\bar{G}}_{BC}\right)-\left[{\left({\bar{G}}_{AB}\right)}^{2}+{\left({\bar{G}}_{AC}\right)}^{2}+{\left({\bar{G}}_{BC}\right)}^{2}\right]\right\}, \end{array}$$

where in the last step we have used $d_{AB}^{2}=-2{\bar{G}}_{AB}$ from Equation (42). Then, taking into account (46), (A1)–(A3), we obtain the relation

(A4) $${\left(6f_{A}f_{B}f_{C}V_{UABC}\right)}^{2}=\Delta_{ABC}+{\left(2f_{A}f_{B}f_{C}A_{ABC}\right)}^{2},$$

that gives (49).

To prove relation (50) of Proposition 3, let us develop the Gramian determinant of a quad of unit vectors $\{n_{A}$, $n_{B}$, $n_{C}$, $n_{D}\}$, which identically vanishes and leads to an angular constraint, $C_{ABCD}=0$, for each quad of emitters. To simplify the notation, let us number the quad from 1 to 4 and use $n_{1}$, $n_{2}$, $n_{3}$, and $n_{4}$ for the respective unit vectors of the emitter positions on $S_{U}$ (forming an inscribed tetrahedron on $S_{U}$, see Figure 2). Consequently,

(A5) $$C_{1234}\equiv \left|\begin{array}{cccc} 1 & n_{1}\cdot n_{2} & n_{1}\cdot n_{3} & n_{1}\cdot n_{4}\\ n_{1}\cdot n_{2} & 1 & n_{2}\cdot n_{3} & n_{2}\cdot n_{4}\\ n_{1}\cdot n_{3} & n_{2}\cdot n_{3} & 1 & n_{3}\cdot n_{4}\\ n_{1}\cdot n_{4} & n_{2}\cdot n_{4} & n_{3}\cdot n_{4} & 1 \end{array}\right|=\left|\begin{array}{cccc} 1 & c_{12} & c_{13} & c_{14}\\ c_{12} & 1 & c_{23} & c_{24}\\ c_{13} & c_{23} & 1 & c_{34}\\ c_{14} & c_{24} & c_{34} & 1 \end{array}\right|=0,$$

which is an algebraic identity involving the the six angles $\theta_{AB}$ defined by pairs of emitters, with A,B=1,2,3,4. The angular identity (A5) is expressed as follows:

(A6) $$\begin{array}{ccc} C_{1234} & = & c_{12}^{2}c_{34}^{2}+c_{13}^{2}c_{24}^{2}+c_{14}^{2}c_{23}^{2}-2\left(c_{12}c_{13}c_{24}c_{34}+c_{12}c_{14}c_{23}c_{34}+c_{13}c_{14}c_{23}c_{24}\right)\\ & & +2(c_{12}c_{13}c_{23}+c_{12}c_{14}c_{24}+c_{13}c_{14}c_{34}+c_{23}c_{24}c_{34})\\ & & -(c_{12}^{2}+c_{13}^{2}+c_{14}^{2}+c_{23}^{2}+c_{24}^{2}+c_{34}^{2})+1=0. \end{array}$$

The inner products of the corresponding quad of light-like vectors $\left\{l_{1},l_{2},l_{3},l_{4}\right\}$ provide a 4 × 4 reduced Gram matrix,

(A7) $${\left(\bar{G}\right)}_{AB}=\left(\begin{array}{cccc} 0 & a & b & c\\ a & 0 & d & e\\ b & d & 0 & f\\ c & e & f & 0 \end{array}\right),$$

where $a\equiv -1+c_{12}$, $b\equiv -1+c_{13}$, $c\equiv -1+c_{14}$, $d\equiv -1+c_{23}$, $e\equiv -1+c_{24}$, and $f\equiv -1+c_{34}$. Substituting these definitions in (A6) and simplifying leads to

(A8) $$\begin{array}{ccc} C_{1234} & = & a^{2}f^{2}+b^{2}e^{2}+c^{2}d^{2}-2\left(abef+acdf+bcde\right)\\ & & +2[a^{2}f+b^{2}e+c^{2}d+af^{2}+be^{2}+cd^{2}+abd+ace+bcf+def\\ & & -(abe+abf+acd+acf+adf+aef+bcd+bce+bde+bef+cde+cdf)]=0.\; \end{array}$$

Notice that $C_{1234}$ is the sum of two homogeneous polynomials $\Delta_{4}$ and $\Delta_{3}$ of fourth and third degree, respectively, in the reduced Gram components (a,b,c,d,e,f). These polynomials are defined as follows:

(A9) $$\begin{array}{cccc} \Delta_{4} & \equiv & \; & \det{\bar{G}}_{4\times 4}=a^{2}f^{2}+b^{2}e^{2}+c^{2}d^{2}-2\left(abef+acdf+bcde\right),\;\\ \Delta_{3} & \equiv & \; & 2[a^{2}f+b^{2}e+c^{2}d+af^{2}+be^{2}+cd^{2}+abd+ace+bcf+def\; \end{array}$$

(A10) $$\begin{array}{c} \;-(abe+abf+acd+acf+adf+aef+bcd+bce+bde+bef+cde+cdf)]. \end{array}$$

Then, for any quad of emitters, angular identity (A8) is expressed in the following equivalent ways:

(A11) $$C_{1234}=0\;⟺\;\Delta_{4}=-\Delta_{3}\;⟺\;\Delta_{1234}=\det{\bar{G}}_{4\times 4}=-{\left(6V_{1234}\right)}^{2}.$$

The last equivalence comes from the mixed product of the triad of relative positions $\left\{n_{1}-n_{2},n_{1}-n_{3},n_{1}-n_{4}\right\}$, the square of such mixed product being equal to its Gram determinant:

(A12) $$\begin{array}{ccc} {\left(6V_{1234}\right)}^{2} & = & {\left(n_{1}-n_{2},n_{1}-n_{3},n_{1}-n_{4}\right)}^{2}\\ & = & \left|\begin{array}{ccc} {\left(n_{1}-n_{2}\right)}^{2} & \left(n_{1}-n_{2}\right)\cdot \left(n_{1}-n_{3}\right) & \left(n_{1}-n_{2}\right)\cdot \left(n_{1}-n_{4}\right)\\ \left(n_{1}-n_{2}\right)\cdot \left(n_{1}-n_{3}\right) & {\left(n_{1}-n_{3}\right)}^{2} & \left(n_{1}-n_{3}\right)\cdot \left(n_{1}-n_{4}\right)\\ \left(n_{1}-n_{2}\right)\cdot \left(n_{1}-n_{4}\right) & \left(n_{1}-n_{3}\right)\cdot \left(n_{1}-n_{4}\right) & {\left(n_{1}-n_{4}\right)}^{2} \end{array}\right|\\ & = & -\left|\begin{array}{ccc} 2a & a+b-d & a+c-e\\ a+b-c & 2b & b+c-f\\ a+c-e & b+c-f & 2c \end{array}\right|, \end{array}$$

where in the last equality, we have substituted the expressions for the squared lengths $d_{AB}^{2}$ of the inscribed tetrahedron edges

(A13) $$\begin{array}{lll} d_{12}^{2}=\;{\left(n_{1}-n_{2}\right)}^{2}=-2a, & \;d_{13}^{2}={\left(n_{1}-n_{3}\right)}^{2}=-2b, & \;d_{14}^{2}={\left(n_{1}-n_{4}\right)}^{2}=-2c, \end{array}$$

(A14) $$\begin{array}{lll} d_{23}^{2}=\;{\left(n_{2}-n_{3}\right)}^{2}=-2d, & \;d_{24}^{2}={\left(n_{2}-n_{4}\right)}^{2}=-2e, & \;d_{34}^{2}={\left(n_{3}-n_{4}\right)}^{2}=-2f, \end{array}$$

and the inner products

(A15) $$\begin{array}{ll} \left(n_{1}-n_{2}\right)\cdot \left(n_{1}-n_{3}\right)=-a-b+d, & \;\left(n_{1}-n_{2}\right)\cdot \left(n_{1}-n_{4}\right)=-a-c+e, \end{array}$$

(A16) $$\begin{array}{c} \left(n_{1}-n_{3}\right)\cdot \left(n_{1}-n_{4}\right)=-b-c+f.\; \end{array}$$

After some algebraic manipulation, the right-hand side of Equation (A12) simplifies to the right-hand side of (A10) and therefore

(A17) $${\left(6V_{1234}\right)}^{2}=\Delta_{3},$$

establishing the second equivalence in (A11). Finally, taking into account that $\Delta_{ABCD}={\left(f_{A}f_{B}f_{C}f_{D}\right)}^{2}\Delta_{1234}$, it is proved that any quad {A,B,C,D} of emitters satisfies (50), that is,

$$\Delta_{ABCD}=-{\left(6f_{A}f_{B}f_{C}f_{D}V_{ABCD}\right)}^{2}.$$

## Appendix B. Proof of Proposition 4

In Proposition 4, item (i) directly follows from (48) and (45) by taking summation over all emitter pairs. To prove (ii), let us start from Equation (A3) using $G_{AB}=f_{A}f_{B}{\bar{G}}_{AB}$,

(A18) $$\begin{array}{cc} {\left(2{f_{A}f_{B}f_{C}A}_{ABC}\right)}^{2}= & \;2(f_{B}f_{C}G_{AB}G_{AC}+f_{A}f_{C}G_{AB}G_{BC}+f_{A}f_{B}G_{AC}G_{BC})\\ & \;-[{\left(f_{C}G_{AB}\right)}^{2}+{\left(f_{B}G_{AC}\right)}^{2}+{\left(f_{A}G_{BC}\right)}^{2}], \end{array}$$

and take summation over all triads of values A,B,C=1,…,k:

(A19) $$\begin{array}{ccc} \sum_{A,B,C=1}^{k}{\left(2f_{A}f_{B}f_{C}A_{ABC}\right)}^{2} & = & 2\sum_{A,B,C=1}^{k}\left(f_{B}f_{C}G_{AB}G_{AC}+f_{A}f_{C}G_{AB}G_{BC}+f_{A}f_{B}G_{AC}G_{BC}\right)\\ & & -\sum_{A,B,C=1}^{k}\left[{\left(f_{C}G_{AB}\right)}^{2}+{\left(f_{B}G_{AC}\right)}^{2}+{\left(f_{A}G_{BC}\right)}^{2}\right]\\ & = & 6\sum_{A,B=1}^{k}f_{A}f_{B}{\left(G^{2}\right)}_{AB}-3K\sum_{A,B=1}^{k}{\left(G_{AB}\right)}^{2}=6\Sigma -3K\sigma , \end{array}$$

which, after dividing by 3!=6, leads to (60). Similarly, to prove (iii), let us start from (A17), replacing numbers (1234) by (ABCD),

(A20) $$\begin{array}{cc} {\left(6f_{A}f_{B}f_{C}f_{D}V_{ABCD}\right)}^{2}= & 2[{\left(G_{AB}\right)}^{2}f_{C}f_{D}G_{CD}+{\left(G_{AC}\right)}^{2}f_{B}f_{D}G_{BD}+{\left(G_{AD}\right)}^{2}f_{B}f_{C}G_{BC}\\ & +f_{A}f_{B}G_{AB}{\left(G_{CD}\right)}^{2}+f_{A}f_{C}G_{AC}{\left(G_{BD}\right)}^{2}+f_{A}f_{D}G_{AD}{\left(G_{BC}\right)}^{2}\\ \; & +f_{B}^{2}G_{AC}G_{AD}G_{CD}+f_{C}^{2}G_{AB}G_{AD}G_{BD}+f_{D}^{2}G_{AB}G_{AC}G_{BC}+f_{A}^{2}G_{BC}G_{BD}G_{CD}\\ \; & -(f_{C}f_{D}G_{AB}G_{AC}G_{BD}+f_{B}f_{D}G_{AB}G_{AC}G_{CD}+f_{C}f_{D}G_{AB}G_{AD}G_{BC}\\ & +f_{B}f_{C}G_{AB}G_{AD}G_{CD}+f_{A}f_{D}G_{AB}G_{BC}G_{CD}+f_{A}f_{C}G_{AB}G_{BD}G_{CD}\\ & +f_{B}f_{D}G_{AC}G_{AD}G_{BC}+f_{B}f_{C}G_{AC}G_{AD}G_{BD}+f_{A}f_{D}G_{AC}G_{BC}G_{BD}\\ & +f_{A}f_{B}G_{AC}G_{BD}G_{CD}+f_{A}f_{C}G_{AD}G_{BC}G_{BD}+f_{A}f_{B}G_{AD}G_{BC}G_{CD})] \end{array}$$

and taking summation over all quads of values A,B,C,D=1,…,k,

(A21) $$\begin{array}{ccc} \sum_{A,B,C,D=1}^{k}{\left(6f_{A}f_{B}f_{C}f_{D}V_{ABCD}\right)}^{2} & = & 2[6\sum_{A,B=1}^{k}{\left(G_{AB}\right)}^{2}\sum_{C,D=1}^{k}f_{C}f_{D}G_{CD}\\ & & +\;4\;K\sum_{A,B,C=1}^{k}G_{AB}G_{AC}G_{BC}-12\sum_{A,B=1}^{k}f_{A}f_{B}{\left(G^{3}\right)}_{AB}]\\ & = & 2\;\left(6\sigma S+4K\lambda -12\Lambda \right)=24\;\left(S\frac{\sigma }{2}+K\frac{\lambda }{3}-\Lambda \right) \end{array}$$

which, after dividing by 4!=24, leads to (61).

## Appendix C. Proof of Proposition 7

The FGDOP matrix, $G^{-1}$, can be computed from the powers of *G* and its traces using (74). In the following, this computation will be carried out by using the tensor expression of *G* given by (38).

### Appendix C.1. Computing $G^{2}$ and tr $G^{2}$

We start by computing the square of *G*:

(A22) $$\begin{array}{cc} G^{2}= & \left[\sum_{A=1}^{k}l_{A}⊗\;{\underset{*}{l}}_{A}-\;2\left(\sum_{B=1}^{k}f_{B}l_{B}\right)⊗\;\underset{*}{u}\right]\times \left[\sum_{C=1}^{k}l_{C}⊗\;{\underset{*}{l}}_{C}-2\left(\sum_{D=1}^{k}f_{D}l_{D}\right)⊗\;\underset{*}{u}\right]\\ = & \sum_{A,C=1}^{k}G_{AC}\;l_{A}⊗\;{\underset{*}{l}}_{C}-2\sum_{A,D=1}^{k}f_{D}G_{AD}\;l_{A}⊗\;\underset{*}{u}\\ & +2\sum_{B,C=1}^{k}f_{B}f_{C}\;l_{B}⊗\;{\underset{*}{l}}_{C}-4\sum_{B,D=1}^{k}f_{B}f_{D}^{2}\;l_{B}⊗\;\underset{*}{u}\;, \end{array}$$

where we have taken into account that $\underset{*}{l_{A}}\times l_{B}={\left(l_{A}\right)}_{\mu }l_{B}^{\mu }=g\left(l_{A},l_{B}\right)=G_{AB}$ and $\underset{*}{u}\times l_{A}=u_{\mu }l_{A}^{\mu }=g\left(u,l_{A}\right)=-f_{A}$. Then, the trace of (A22) is

(A23) $$\begin{array}{ccc} \beta \equiv \mathrm{tr}G^{2} & = & \sum_{A,C=1}^{k}{\left(G_{AC}\right)}^{2}+2\sum_{A,D=1}^{k}f_{A}f_{D}\;G_{AD}+2\sum_{B,C=1}^{k}f_{B}f_{C}\;G_{BC}+4\sum_{B,D=1}^{k}f_{B}^{2}f_{D}^{2}\\ & = & \sigma +4S+4K^{2}, \end{array}$$

where the last step follows after summation and using the definitions given in (55) and (56) for *σ* and *S*, respectively.

### Appendix C.2. Computing $G^{3}$ and tr $G^{3}$

Now, following a similar calculation, the product of (A22) and (38) gives

(A24) $$\begin{array}{ccc} G^{3} & = & \left[\sum_{A,C=1}^{k}G_{AC}\;l_{A}⊗\;{\underset{*}{l}}_{C}-2\sum_{A,D=1}^{k}f_{D}G_{AD}\;l_{A}⊗\;\underset{*}{u}+2\sum_{B,C=1}^{k}f_{B}f_{C}\;l_{B}⊗\;{\underset{*}{l}}_{C}-4\sum_{B,D=1}^{k}f_{B}f_{D}^{2}\;l_{B}⊗\;\underset{*}{u}\right]\;\\ & & \times \left[\sum_{E=1}^{k}l_{E}⊗\;{\underset{*}{l}}_{E}-2\left(\sum_{E=1}^{k}f_{E}l_{E}\right)⊗\;\underset{*}{u}\right]\\ & = & \sum_{A,C,E=1}^{k}G_{AC}G_{CE}\;l_{A}⊗\;{\underset{*}{l}}_{E}-\;2\sum_{A,C,E=1}^{k}f_{E}G_{AC}G_{CE}\;l_{A}⊗\;\underset{*}{u}\\ & & +\;2\sum_{A,D,E=1}^{k}f_{E}f_{D}G_{AD}\;l_{A}⊗\;{\underset{*}{l}}_{E}-\;4\sum_{A,D,E=1}^{k}f_{E}^{2}f_{D}G_{AD}\;l_{A}⊗\;\underset{*}{u}\\ & & +\;2\sum_{B,C,E=1}^{k}f_{B}f_{C}G_{CE}\;l_{B}⊗\;{\underset{*}{l}}_{E}-\;4\sum_{B,C,E=1}^{k}f_{B}f_{C}f_{E}G_{CE}\;l_{B}⊗\;\underset{*}{u}\\ & & +\;4\sum_{B,D,E=1}^{k}f_{B}f_{D}^{2}f_{E}\;l_{B}⊗\;{\underset{*}{l}}_{E}-\;8\sum_{B,D,E=1}^{k}f_{B}f_{D}^{2}f_{E}^{2}\;l_{B}⊗\;\underset{*}{u}. \end{array}$$

Then, taking the trace,

(A25) $$\begin{array}{ccc} \gamma \equiv \mathrm{tr}G^{3} & = & \sum_{A,C,E=1}^{k}G_{AC}G_{CE}G_{AE}+2\sum_{A,C,E=1}^{k}f_{A}f_{E}\;G_{AC}G_{CE}\\ & & +2\sum_{A,D,E=1}^{k}f_{E}f_{D}G_{AD}G_{AE}+4\sum_{A,D,E=1}^{k}f_{E}^{2}f_{A}f_{D}G_{AD}\\ & & +2\sum_{B,C,E=1}^{k}f_{C}f_{D}G_{CE}G_{BE}+4\sum_{B,C,E=1}^{k}f_{C}f_{B}^{2}f_{E}G_{CE}\\ & & +4\sum_{B,D,E=1}^{k}f_{B}f_{D}^{2}f_{E}^{2}G_{BE}+8\sum_{B,D,E=1}^{k}f_{B}^{2}f_{D}^{2}f_{E}^{2}\\ & = & \sum_{A,B,C=1}^{k}G_{AB}G_{BC}G_{CA}+6\sum_{A,B,C=1}^{k}f_{A}f_{B}G_{AB}G_{BC}\\ & & +12K\sum_{A,B=1}^{k}f_{A}G_{AB}+8\sum_{A,B,C}^{k}f_{A}^{2}f_{B}^{2}f_{C}^{2}\\ & = & \lambda +6\Sigma +12KS+8K^{3}. \end{array}$$

where the last step follows after summation and using the definitions given in (55), (57) and (56) for *λ*, Σ, and *S*, respectively.

### Appendix C.3. Computing $G^{4}$ and tr $G^{4}$

The fourth power of *G* is computed in a similar way by extending the above procedure:

(A26) $$\begin{array}{ccc} G^{4} & = & G^{3}\times \left[\sum_{F=1}^{k}l_{F}⊗\;{\underset{*}{l}}_{F}-2\left(\sum_{H=1}^{k}f_{H}l_{H}\right)⊗\;\underset{*}{u}\right]\\ & = & \sum_{A,C,E,F=1}^{k}G_{AC}G_{CE}G_{EF}\;l_{A}⊗\;{\underset{*}{l}}_{F}-\;2\sum_{A,C,E,H=1}^{k}f_{H}G_{AC}G_{CE}G_{EH}\;l_{A}⊗\;\underset{*}{u}\\ & & \;+2\sum_{A,C,E,F=1}^{k}f_{E}f_{F}G_{AC}G_{CE}\;l_{A}⊗\;{\underset{*}{l}}_{F}-\;4\sum_{A,C,E,H=1}^{k}f_{E}f_{H}^{2}G_{AC}G_{CE}\;l_{A}⊗\;\underset{*}{u}\\ & & +\;2\sum_{A,D,E,F=1}^{k}f_{E}f_{D}G_{AD}G_{EF}\;l_{A}⊗\;{\underset{*}{l}}_{F}-\;4\sum_{A,D,E,H=1}^{k}f_{E}f_{D}f_{H}G_{AD}G_{EH}\;l_{A}⊗\;\underset{*}{u}\\ & & +\;4\sum_{A,D,E,F=1}^{k}f_{E}^{2}f_{F}f_{D}G_{AD}\;l_{A}⊗\;{\underset{*}{l}}_{F}-\;8\sum_{A,D,E,H=1}^{k}f_{E}^{2}f_{H}^{2}f_{D}G_{AD}\;l_{A}⊗\;\underset{*}{u}\\ & & +\;2\sum_{B,C,E,F=1}^{k}f_{C}f_{B}G_{CE}G_{EF}\;l_{B}⊗\;{\underset{*}{l}}_{F}-\;4\sum_{B,C,E,H=1}^{k}f_{C}f_{B}f_{H}G_{CE}G_{EH}\;l_{B}⊗\;\underset{*}{u}\\ & & +\;4\sum_{B,C,E,F=1}^{k}f_{B}f_{C}f_{E}f_{F}G_{CE}\;l_{B}⊗\;{\underset{*}{l}}_{F}-\;8\sum_{B,C,E,H=1}^{k}f_{B}f_{C}f_{E}f_{H}^{2}G_{CE}\;l_{B}⊗\;\underset{*}{u}\\ & & +\;4\sum_{B,D,E,F=1}^{k}f_{B}f_{D}^{2}f_{E}G_{EF}\;l_{B}⊗\;{\underset{*}{l}}_{F}-\;8\sum_{B,D,E,H=1}^{k}f_{B}f_{D}^{2}f_{E}f_{H}G_{EH}\;l_{B}⊗\;\underset{*}{u}\\ & & +\;8\sum_{B,D,E,F=1}^{k}f_{B}f_{D}^{2}f_{E}^{2}f_{F}\;\;l_{B}⊗\;{\underset{*}{l}}_{F}-\;16\sum_{B,D,E,H=1}^{k}f_{B}f_{D}^{2}f_{E}^{2}f_{H}^{2}\;\;l_{B}⊗\;\underset{*}{u}. \end{array}$$

Renaming summation indexes and grouping conveniently, we obtain the following expression:

(A27) $$\begin{array}{ccc} G^{4} & = & \sum_{A,B,C,D=1}^{k}G_{AB}G_{BC}G_{CD}\;l_{A}⊗\;{\underset{*}{l}}_{D}-\;2\sum_{A,B,C,D=1}^{k}f_{D}G_{AB}G_{BC}G_{CD}\;l_{A}⊗\;\underset{*}{u}\\ & & \;+2\sum_{A,B,C=1}^{k}f_{C}G_{AB}G_{BC}\;l_{A}⊗\left(\sum_{D=1}^{k}f_{D}{\underset{*}{l}}_{D}\right)-\;4K\sum_{A,B,C=1}^{k}f_{C}G_{AB}G_{BC}\;l_{A}⊗\;\underset{*}{u}\\ & & +\;2\sum_{A,B,C,D=1}^{k}f_{C}f_{B}G_{AB}G_{CD}\;l_{A}⊗\;{\underset{*}{l}}_{D}-\;4\sum_{A,B,C,D=1}^{k}f_{B}f_{C}f_{D}G_{AB}G_{CD}\;l_{A}⊗\;\underset{*}{u}\\ & & +\;4K\left(\sum_{A,B}^{k}f_{B}G_{AB}\;l_{A}\right)⊗\sum_{C=1}^{k}f_{C}{\underset{*}{l}}_{C}-\;8K^{2}\sum_{A,B=1}^{k}f_{B}G_{AB}\;l_{A}⊗\;\underset{*}{u}\\ & & +\;2\sum_{A,B,C,D=1}^{k}f_{B}G_{BC}G_{CD}\;f_{A}\;l_{A}⊗\;{\underset{*}{l}}_{D}-\;4\sum_{A,B,C,D=1}^{k}f_{B}f_{A}f_{D}G_{BC}G_{CD}\;l_{A}⊗\;\underset{*}{u}\\ & & +\;4\left(\sum_{A,B=1}^{k}f_{A}f_{B}G_{AB}\right)\left(\sum_{C=1}^{k}\;f_{C}\;l_{C}\right)⊗\left(\sum_{D=1}^{k}f_{D}\;{\underset{*}{l}}_{D}\right)\\ & & -\;8K\left(\sum_{A,B=1}^{k}f_{A}f_{B}G_{AB}\right)\left(\sum_{C=1}^{k}\;f_{C}\;l_{C}\right)⊗\;\underset{*}{u}+\;4K\left(\sum_{A=1}^{k}f_{A}\;l_{A}\right)⊗\sum_{B,C=1}^{k}f_{B}G_{BC}{\underset{*}{l}}_{C}\\ & & -\;8K\left(\sum_{A,B=1}^{k}f_{A}f_{B}G_{AB}\right)\left(\sum_{C=1}^{k}f_{C}\;l_{C}\right)⊗\;\underset{*}{u}+\;8K^{2}\sum_{A,B=1}^{k}f_{A}f_{B}l_{A}⊗\;{\underset{*}{l}}_{B}\\ & & -\;16K^{3}\left(\sum_{A=1}^{k}f_{A}\;l_{A}\right)⊗\;\underset{*}{u}. \end{array}$$

And then, taking the trace of (A27), we obtain

(A28) $$\begin{array}{ccc} \delta \equiv \mathrm{tr}G^{4} & = & \sum_{A,B,C,D=1}^{k}G_{AB}G_{BC}G_{CD}G_{DA}+\;2\sum_{A,B,C,D=1}^{k}f_{A}f_{D}G_{AB}G_{BC}G_{CD}\\ & & +2\sum_{A,B,C,D=1}^{k}f_{C}f_{D}G_{AB}G_{BC}G_{AD}+4K\sum_{A,B,C=1}^{k}f_{A}f_{C}G_{AB}G_{BC}\\ & & +2\sum_{A,B,C,D=1}^{k}f_{C}f_{B}G_{AB}G_{CD}G_{AD}+4{\left(\sum_{A,B=1}^{k}f_{A}f_{B}G_{AB}\right)}^{2}\\ & & +4K\sum_{A,B,C=1}^{k}f_{B}f_{C}G_{AB}G_{AC}+\;8K^{2}\sum_{A,B=1}^{k}f_{A}f_{B}G_{AB}\\ & & +2\sum_{A,B,C,D=1}^{k}f_{B}f_{A}G_{BC}G_{CD}G_{DA}+4K\sum_{A,B,C=1}^{k}f_{A}f_{C}G_{AB}G_{BC}\\ & & +4{\left(\sum_{A,B=1}^{k}f_{A}f_{B}G_{AB}\right)}^{2}+8K^{2}\sum_{A,B=1}^{k}f_{A}f_{B}G_{AB}\\ & & +4K\sum_{A,B,C=1}^{k}f_{A}f_{B}G_{BC}G_{CA}+\;8K^{2}\sum_{A,B=1}^{k}f_{A}f_{B}G_{AB}\\ & & +8K^{2}\sum_{A,B=1}^{k}f_{A}f_{B}G_{AB}+16K^{4}\\ & = & \omega +8\Lambda +8S^{2}+16K\Sigma +32K^{2}S+16K^{4}, \end{array}$$

where the last step follows after summation and using for *ω*, *S*, Σ, and Λ the definitions given in (55), (56), (57), and (58), respectively.
